# G-quadruplex ligand RHPS4 radiosensitizes glioblastoma xenograft in vivo through a differential targeting of bulky differentiated- and stem-cancer cells

**DOI:** 10.1186/s13046-019-1293-x

**Published:** 2019-07-16

**Authors:** F. Berardinelli, M. Tanori, D. Muoio, M. Buccarelli, A. di Masi, S. Leone, L. Ricci-Vitiani, R. Pallini, M. Mancuso, A. Antoccia

**Affiliations:** 1 Department of Science, University Roma Tre, Rome, Italy; 2 Laboratory of Biomedical Technologies, Agenzia Nazionale per le Nuove Tecnologie, l’Energia e lo Sviluppo Economico Sostenibile (ENEA), Rome, Italy; 3 Department of Oncology and Molecular Medicine, Istituto Superiore di Sanità, Rome, Italy; 4 Institute of Neurosurgery, Università Cattolica del Sacro Cuore, Fondazione Policlinico Universitario Agostino Gemelli, Rome, Italy

**Keywords:** G4 ligands, RHPS4, Telomeres, Radiosensitization, Glioma stem-like cells

## Abstract

**Background:**

Glioblastoma is the most aggressive and most lethal primary brain tumor in the adulthood. Current standard therapies are not curative and novel therapeutic options are urgently required. Present knowledge suggests that the continued glioblastoma growth and recurrence is determined by glioblastoma stem-like cells (GSCs), which display self-renewal, tumorigenic potential, and increased radio- and chemo-resistance. The G-quadruplex ligand RHPS4 displays in vitro radiosensitizing effect in GBM radioresistant cells through the targeting and dysfunctionalization of telomeres but RHPS4 and Ionizing Radiation (IR) combined treatment efficacy in vivo has not been explored so far.

**Methods:**

RHPS4 and IR combined effects were tested in vivo in a heterotopic mice xenograft model and in vitro in stem-like cells derived from U251MG and from four GBM patients. Cell growth assays, cytogenetic analysis, immunoblotting, gene expression and cytofluorimetric analysis were performed in order to characterize the response of differentiated and stem-like cells to RHPS4 and IR in single and combined treatments.

**Results:**

RHPS4 administration and IR exposure is very effective in blocking tumor growth in vivo up to 65 days. The tumor volume reduction and the long-term tumor control suggested the targeting of the stem cell compartment. Interestingly, RHPS4 treatment was able to strongly reduce cell proliferation in GSCs but, unexpectedly, did not synergize with IR. Lack of radiosensitization was supported by the GSCs telomeric-resistance observed as the total absence of telomere-involving chromosomal aberrations. Remarkably, RHPS4 treatment determined a strong reduction of CHK1 and RAD51 proteins and transcript levels suggesting that the inhibition of GSCs growth is determined by the impairment of the replication stress (RS) response and DNA repair.

**Conclusions:**

We propose that the potent antiproliferative effect of RHPS4 in GSCs is not determined by telomeric dysfunction but is achieved by the induction of RS and by the concomitant depletion of CHK1 and RAD51, leading to DNA damage and cell death. These data open to novel therapeutic options for the targeting of GSCs, indicating that the combined inhibition of cell-cycle checkpoints and DNA repair proteins provides the most effective means to overcome resistance of GSC to genotoxic insults.

**Electronic supplementary material:**

The online version of this article (10.1186/s13046-019-1293-x) contains supplementary material, which is available to authorized users.

## Background

Glioblastoma Multiforme (GBM) is an aggressive and very heterogeneous tumor of the central nervous system. GBM is one of the most lethal type of tumors, whose heterogeneous features render its management very difficult [1], as demonstrated by the 5-year survival of less than 5% of patients [2]. GBM is typically treated by a combination of surgical resection, radiotherapy and chemotherapy with temozolomide (TMZ). In general, the poor patient survival is due to the recurrence of GBM despite therapy [3, 4]. It has been suggested that the unavoidable recurrence is driven by a subpopulation of GBM cells with stem cell properties known as glioblastoma stem-like cells (GSCs) or glioblastoma initiating cells (GICs) [5]. Indeed, GSCs have exclusive features as self-renewal, tumorigenic potential in vivo, and increased chemo- and radio-resistance, which are thought to be one of the main reasons of GBM poor prognosis. Upregulated DNA damage response (DDR) has been documented in GSCs, including enhanced checkpoint signaling and hyperactivation of repair proteins such as ATM, CHK1, CHK2 and RAD51 [6–10]. In particular, homologous recombination (HR), rather than non homologous end joining (NHEJ), is the preferential pathway involved in DNA double strand breaks (DSBs) repair in GSCs, pointing to the targeting of RAD51 as a possible strategy to overcome the intrinsic radioresistance of these cells [6, 11–13]. In addition to DNA repair proteins, telomeric targeting is a further attractive strategy to sensitize glioma radioresistant cells to conventional radiotherapy and to adrontherapy [14–17].

Telomeres are nucleoprotein structures located at the ends of linear eukaryotic chromosomes, composed by tandem repetition of the TTAGGG hexanucleotide [18]. In physiological conditions, telomeres (but also other G-rich regions) are capable to assume non-canonical DNA helical structures known as G-quadruplex (G4) [19]. The stabilization of telomeric G4 structures represents one of the most effective strategies among the approaches proposed to specifically target telomeres. This purpose can be achieved by using small molecules that bind and stabilize G4, such as the 3,11-difluoro-6,8,13-trimethylquino [4,3,2-kl]acridinium methylsulfate (RHPS4) [20]. This drug binds principally to G4 in telomeric regions causing telomeric damage, cell cycle arrest, and cell growth impairment. In addition, RHPS4 can target G4 structures located in promoters and introns of a set of genes involved in cancer progression (e.g., *MYC*, *VEGFR2*) and stemness (e.g., *CD133*, *CD44*) [21].

The RHPS4 anti-proliferative activity has been characterized in various in vitro and in vivo tumor models [20, 22, 23] and, in addition, our group demonstrated that RHPS4 is also a powerful radiosensitizing agent [16, 17]. The mechanism by which RHPS4 radiosensitizes GBM cells to both low- and high-LET ionizing radiations (IR) is related to its ability to target telomeres, thus making them dysfunctional and increasing recombinogenic chromosome ends that interfere with correct repair of IR-induced DSBs [16, 24]. As a result, this increases the rate of lethal chromosome aberrations that involve telomeres (e.g., telomere-telomere and telomere-DSB fusions), which in turn led to cell death.

Here, we tested the radiosensitizing power of RHPS4 in a mice heterotopic GBM-xenograft model derived from U251MG cells as well as, in two different in vitro cellular models of GSCs (i.e., stem-like cells obtained from U251MG and patient-derived GSCs). Results obtained indicated that RHPS4 is very powerful in radiosensitizing GBM in vivo, although differentiated cancer cells and GSCs respond differently to the compound. In particular, in both the GSCs models, RHPS4 was very effective in blocking cell proliferation but, differently from differentiated cells [16, 17], it failed to induce telomeric damage and radiosensitization. Our data suggest that the potent antiproliferative effect exerted by RHPS4 in GSCs is determined by strong reduction of RAD51 and CHK1 with consequent replicative stress and cell cycle blockage.

## Materials and methods

### Chemical compound

The 3,11-difluoro-6,8,13-trimethylquino [4,3,2-kl]acridinium methylsulfate (RHPS4) (Tocris Bioscience, Bristol, UK) was dissolved in dimethyl sulfoxide (DMSO) at a stock concentration of 10 mM solution for the in vitro use. In all the in vitro experiments, RHPS4 was added to the cell medium at least 8 h after plating. An appropriate volume of DMSO was employed as negative control. For the in vivo study, RHPS4 was dissolved in PBS and administered intravenously (10 mg/kg per day) in immunocompromised mice. PBS only was administrated as negative control. Drug dilutions were freshly prepared periodically before each set of experiments.

### Cell lines and culture conditions

Unless otherwise specified, media and supplements for cell culture were purchased from Euroclone (Euroclone, Pero, Milan, Italy) and the plasticware was purchased from Corning (Corning Life Sciences, NY, USA). U251MG cell line was purchased from Banca Biologica and Cell Factory (Banca Biologica and Cell Factory, Genoa, Italy). The U251MG cell line (here named U251MG-Adh) was routinely maintained in Minimum Essential Medium with Earle’s Balanced Salt Solution (MEM/EBSS) supplemented with 10% fetal bovine serum (FBS), 2 mM L-glutamine, 1 mM sodium Pyruvate (Euroclone), 1% non-essential aminoacids, 100 units/mL penicillin and 100 μg/mL streptomycin. For neurosphere generation (here named U251MG-Sph), the U251MG cell line was cultured in NeuroCult™ Proliferation NS-A Basal Medium (Stemcell Technologies, Vancouver, Canada), complemented with NeuroCult™ NS-A Proliferation Supplement (Stemcell Technologies), 20 ng/ml of recombinant human epidermal growth factor (EGF) (Stemcell Technologies), 10 ng/mL of basic fibroblast growth factor (b-FGF) (Stemcell Technologies) and 2 μg/mL heparin solution (Stemcell Technologies).

GSCs were obtained from adult GBM patients (WHO grade IV), who had undergone complete or partial surgical resection at the Institute of Neurosurgery, Catholic University School of Medicine, Rome, Italy. Informed consent was obtained from the patients before surgery. The tumor tissue was mechanically dissociated and single cell suspension was cultured in a serum-free medium supplemented with epidermal growth factor and basic fibroblast growth factor, as previously described [25–27]. The in vivo tumorigenic potential of GSCs was evaluated by intracranial cell injection in immunocompromised mice, where GSCs were able to recapitulate the patient tumor in terms of antigen expression and histological tissue organization. Packaging human embryonic kidney cell line 293 T was maintained in DMEM (Euroclone) supplemented with 10% (v/v) heat-inactivated FBS, 2 mM L-glutamine, 100 U/ml penicillin and 100 μg/ml streptomycin (Invitrogen, Life Technologies, Carlsbad, CA, USA).

All the aforementioned cell lines were maintained at 37 °C in a 5% CO_2_ 95% air atmosphere.

### Irradiation conditions and combined treatments

U251MG-Sph cells were irradiated with X-ray at room temperature (RT) using a Gilardoni apparatus (250 kV, 6 mA; Gilardoni S.p.A., Mandello del Lario, Lecco, Italy) operating at a dose rate of 0.53 Gy/min (University Roma Tre X-rays facility). Cells were seeded as monocellular suspension, treated with RHPS4 (0.2, 0.5, and 1 μM) and after 5 days exposed to 10 Gy X-rays. GSCs were exposed to a single dose of acute cesium-137 (137Cs) γ-irradiation operating at a dose rate of 0.8 Gy/min (Istituto Superiore Sanità facility). Combined treatments were performed by treating cells for 4 days with RHPS4 (concentrations used depending on the cell line and type of experiment) and then irradiating them with 10 Gy γ-rays. Cell viability was assessed 3 and 7 days after irradiation.

Mice irradiation was performed using a Gilardoni CHF 320 G X-ray generator (250 kVp, 15 mA; Gilardoni S.p.A., Mandello del Lario, Lecco, Italy) with filters of 2.0 mm Al and 0.5 mm Cu (HVL = 1.6 mm Cu), operating at a dose rate of 0.89 Gy/min) (ENEA Casaccia facility). Combined treatments were performed following different procedures accordingly with the different biological models used (Table 1).

**Table 1:** Combined RHSP4 and irradiation treatment protocols

Target (biological model)	RHPS4 administration	IR exposure
Xenograft GBM tumors in mice	Intravenous injection in caudal vein of 10 mg/kg/die RHPS4 for 5 days or 10 days	At day 5 or 10 tumors were exposed to 10 Gy of X-rays (mice were shielded using appropriate lead plates in order to irradiate only the tumor mass)
U251MG-Adh or U251MG-SC-Sph	RHPS4 0.2, 0.5 or 1 μM was added in the culture medium and maintained for 5 days	At day 5 cells were exposed to 10 Gy of X-rays (250 kV, 6 mA) with a dose rate of 0.53 Gy/min
Patient-derived GSCs	RHPS4 was added in the culture medium and maintained for 4 or 8 days	At day 4 cells were exposed to 10 Gy of γ-rays (cesium-137 γ-irradiation) with a dose rate of 0.8 Gy/min

### Subcutaneous xenograft model

Animal studies were performed according to the European Community Council Directive 2010/63/EU, approved by the local Ethical Committee for Animal Experiments of the ENEA, and authorized by the Italian Ministry of Health (n° 690/2015-PR). Female (*n* = 30) athymic mice (CD1 nude, Charles River, Lecco, Italy) were housed in sterilized filter-topped cages kept in laminar flow isolators, fed with autoclaved food and water ad libitum, and maintained in 12-h light/dark cycle. At 5-weeks of age all mice received 0.25 ml subcutaneous injection of 7.5 × 10^6^ U251MG cells in 50% Matrigel (BD Biosciences, San Diego, CA) into one or both flanks. Inoculated animals were daily monitored and tumors measured with a caliper three times a week. Tumor dimension was estimated using the following formula:

$$ Tumor\ volume=\left( length\times {width}^2\right)/ 2 $$

When tumor mass reached the volume of 800 mm^3^, mice were randomized in four experimental groups: vehicle, RHPS4 (mice with double tumor mass), vehicle + 10 Gy and RHPS4 + 10 Gy groups (mice with single tumor mass). RHPS4 (10 mg/kg per day) or PBS (vehicle) were administered through intravenous injection for 5 days, then mice were irradiated with a single dose of 10 Gy of X-rays. During the delivery time of 10 Gy, mice were lightly anesthetized with 35 mg/Kg of pentobarbital sodium and the body was shielded with 4 mm thick lead plates in order to irradiate only the tumor mass. After treatments, mice were daily monitored and tumor dimension recorded as described above. To evaluate differences in efficacy between treatment groups, the percentage of tumor growth inhibition (TGI) was calculated as follows:

$$ TGI\ \left(\%\right)=\left[\left({Vc}_1\hbox{-} {Vt}_1\right)/\left({Vc}_0\hbox{-} {Vt}_0\right)\right]\times 100 $$

where Vc_1_ and Vt_1_ are the median of control and treated groups at the end of the study, respectively and Vc_0_ and Vt_0_ are the median of control and treated groups at the start of the study [28]. At necropsy, all tumors were removed and collected for histology and immunoblot analysis.

### Immunostaining of 53BP1 on tumor frozen sections

Tumor masses were fixed in 10% neutral buffered formalin at RT for 24 h and immersed in 30% sucrose/PBS at 4 °C twice, until they were sunk. They were embedded in OCT and stored at − 80 °C. Sections were cut at 10 μm and cells were permeabilized with 0.5% Triton X-100 and blocked in 1% BSA/PBS. Samples were then immune-stained overnight (ON) at 4 °C using a rabbit anti-53BP1 antibody (Novus Biologicals, Centennial, CO, USA). After washes in 1% Bovine Serum Albumin (BSA) dissolved in PBS, samples were incubated with the anti-rabbit Alexa 488 secondary antibody (Invitrogen) for 1 h at 37 °C. Finally, slides were washed in 1% BSA/PBS, counterstained with 4,6-diamidino-2-phenylindole (DAPI; Sigma Aldrich, St. Louis, MO) and analyzed by fluorescence microscopy using an Axio-Imager Z2 microscope equipped with a coupled charged device (CCD) camera (Zeiss, Jena, Germany). The frequency of DNA damage marker foci and colocalization dots per cell were scored in 100 nuclei in at least two independent experiments.

### Real time reverse transcription PCR (qRT-PCR)

Total RNA was extracted using TRIzol® (Life Technologies, Carlsband, CA, USA) according to the manufacturer’s instructions. RNA was reverse transcribed using an oligo-dT primer to prime the reverse transcription and the SuperScript™ II Reverse Transcriptase system (Invitrogen). Gene expression levels were analyzed using the SYBR Green PCR Master Mix (Biorad, California, USA). The reaction was performed using the Agilent AriaMx real-time PCR system (Agilent Technologies, California, USA) using the following thermal cycling conditions: 95 °C for 2 min followed by 30 cycles at 95 °C for 10 s and 60 °C for 30 s. The PCR primer sequences were reported in Table 2. Data were reported as relative quantity (RQ) with respect to a calibrator sample (i.e., actin) according to the 2^-ΔΔCt^ method.

**Table 2:** PCR primer sequences

Gene	Forward	Reverse
*SOX2*	5′-GGCAGCTACAGCATGATGCAGGAGC-3′	5′-CTGGTCATGGAGTTGTACTGCAGG-3′
*CD44*	5′-CCACGTGGAGAAAAATGGTC-3′	5′-CATTGGGCAGGTCTGTGAC-3′
*GFAP*	5′-GTGGGCAGGTGGGAGCTTGATCT-3′	5′-CTGGGGCGGCCTGGTATGACA-3′
*NESTIN*	5′-AGGATGTGGAGGTAGTGAGA-3′	5′-TGGAGATCTCAGTGGCTCTT-3′
*RAD51*	5′-GCATAAATGCCAACGATGTG-3’	5′-TTAGCTGCCTCAGCCAGAAT-3’
*CHK1*	5′-CGGTGGAGTCATGGCAGTGCCC-3’	5′-TCTGGACAGTCTACGGCACGCTTCA-3’
*ACTIN*	5′-AGAGGGAAATCGTGCGTGAC-3’	5′-CAATGGTGATGACCTGGCCG-3’

### Western blot

U251MG cells were lysed in 20 mM Tris HCl pH 7.5, 150 mM NaCl, 1 mM EDTA, 1% Triton-100X, and protease inhibitors. Protein extracts (20–30 μg) were loaded on an SDS-PAGE and transferred onto a polyvinylidene fluoride (PVDF) membrane (pore size 0.45 μm; Immobilion-P, Millipore, Massachusetts, USA). Filters were blocked with 3% BSA dissolved in Tris Buffered Saline (TBS) with 0.05% Tween-20 (TBS-T) for 0.5 h at RT. Membranes were then incubated at 4 °C ON with the following primary antibodies: actin β (#A2066, Sigma-Aldrich); ATM (#sc-23,921, Santa Cruz Biotechnology, Dallas, TX, USA); ATR (#sc-515173C1; Santa Cruz Biotechnology); CD44 (#550989, BD Pharmingen, San Josè, CA, USA); CHK1 (#sc-8408, Santa Cruz Biotechnology); CHK2 (#sc-17747A11, Santa Cruz Biotechnology); GFAP (#Z0334, DAKO, Santa Clara, CA, USA); Ku80 (#2180, Cell Signaling, Leiden, The Netherlands); Nestin (#NBP1–02419, Novus Biologicals); PCNA (#sc-56PC10, Santa Cruz Biotechnology); phThr1989-ATR (#58014S, Cell Signaling); phSer345-CHK1 (#2341S, Cell Signaling); phThr68-CHK2 (#2661S, Cell Signaling); phSer1981-ATM (#5883S, Cell Signaling); RAD51 (#sc-33,626; Santa Cruz Biotechnology); SOX2 (# ab97959, Abcam, Cambridge, UK); vinculin (#AB_10976821, Invitrogen). Finally, membranes were incubated 1 h at room temperature with the appropriate HRP-conjugated secondary antibody (Bio-Rad Laboratories, Hercules, CA, USA). Proteins were visualized by the enhanced chemiluminescence detection system. Experiments were repeated at least three times. The images were analyzed with ImageJ.

### Lentiviral infection

For CHK1 silencing experiments in GSC #163, GIPZ™ non-targeting lentiviral shRNA control (NTC) and GIPZ™ CHK1 shRNA (#RHS4531-clone B5; #RHS4531-clone E1; #RHS4531-clone F11) were purchased by Dharmacon (Dharmacon Inc., Lafayette, CO, USA). Lentiviral particles were produced by the calcium phosphate transfection protocol in the packaging cell line 293 T, as previously described [29]. Briefly, the lentiviral construct was cotransfected with pMDL, pRSV-REV and pVSV-G. The calcium-phosphate DNA precipitate was removed after 8 h by replacing the medium. Upon 48 h, viral supernatants were collected and filtered through a 0.45 μm pore size filter and then added to GSCs in the presence of 8 μg/ml polybrene. Cells were centrifuged for 30 min at 1800 rpm. After infection, transduced cells were selected with puromycin (Sigma-Aldrich) and Green Fluorescence Protein (GFP) was evaluated by FACSCanto (BD Biosciences, Milan, Italy).

### Neurospheres cell growth assay

The ability of RHPS4 to reduce the proliferation of U251MG-Sph was evaluated in neurosphere growth-assay. U251MG cells were harvested with trypsin-EDTA when they were in exponential growth, counted and washed in PBS. After removal of PBS, cells were plated in quintuplicate at a density of 3000 cells/well in a non-tissue culture coated 24-well plates (Corning-Costar; Lowell, MA), and treated with increasing concentrations of RHPS4 (i.e., 0.2, 0.5, and 1 μM). After 5 days, cells were exposed to 10 Gy of X-rays and then incubated for additional 5 days. At day 10, images of spheres were captured using an Axiovert 40C microscope (Zeiss) equipped with a Tucsen IS500 camera (Fuzhou Tucsen photonics, China). Analysis of the sphere number and size were performed using the ISCapture 3.0 software (Fuzhou Tucsen photonics, China). Data shown represent the mean of three independent experiments.

### Metaphase spreads preparation

Chromosome spreads were obtained following standard procedures. Briefly, colchicine 5 × 10^− 6^ M was added to the cells 4 h before the finalization of the culture. Cells were then incubated with 75 mM KCl hypotonic solution for 20 min at 37 °C, and subsequently fixed in freshly prepared Carnoy solution (3:1 methanol/acetic acid (v/v)). Cells were then dropped onto slides, air dried, and utilized for cytogenetic analysis.

### Multicolor FISH (M-FISH)

Fixed cells were dropped onto glass slides and hybridised with the 24XCyte Human Multicolour FISH Probe Kit (MetaSystems, Altlussheim, Germany), following the manufacturer’s instructions. Briefly, the slides were denatured in 0.07 N NaOH and then rinsed in a graded ethanol series. Meanwhile, the probe mix was denatured using a MJ mini personal thermal cycler (Bio-Rad) with the following program: 5 min at 75 °C, 30 sec at 10 °C, and 30 min at 37 °C. The probe was added to the slides and a coverslip was added and sealed using rubber cement. The samples were then hybridized in a humidified chamber at 37 °C for 48 h, washed in saline-sodium citrate (SSC) buffer for 5 min at 75 °C, and finally counterstained with DAPI. Metaphases were visualised and captured using an Axio-Imager M1 microscope (Zeiss). The karyotyping and cytogenetic analysis of each single chromosome was performed using the ISIS software (MetaSystems).

### Quantitative telomeric FISH and Pancentromeric and telomeric FISH

Centromere calibrated Q-FISH staining was performed as previously described [30]. Briefly, 48 h after the seeding, slides were rinsed with PBS pH 7.5, and fixed in 4% formaldehyde for 2 min. After two rinses in PBS, slides were incubated in acidified pepsin solution for 10 min, rinsed, and dehydrated through graded alcohols. Slides and probes were co-denatured at 80 °C for 3 min and hybridized for 2 h at room temperature in a humidified chamber (probes were reported in Table 3). After hybridization, slides were washed twice for 15 min in 70% formamide, 10 mM Tris-HCl at pH 7.2, and 0.1% BSA, followed by three 5-min washes in TBS/Tween 20 0.08%. Slides were then dehydrated with an ethanol series and air-dried. Finally, samples were counterstained with DAPI in Vectashield (Vector Laboratories, Burlingame, CA, USA). Images were captured at 63× magnification with an Axio Imager Z2 (Carl Zeiss, Germany) equipped with a charge-coupled device camera, and the telomere size was analyzed with ISIS software (MetaSystems). The software calculates telomere lengths as the ratio between the fluorescence of each telomere signal and the fluorescence of the centromere of chromosome 2, used as the internal reference in each metaphase analyzed. The centromere 2 DNA sequence, which the probe recognizes, has a stable length and can be used as a reference. Data were expressed as a percentage (T/C %) [31, 32]. For each individual, at least 30 metaphases have been analyzed in two independent experiments.

**Table 3:** Probes used in FISH experiments

Telomeric Q-FISH		
Target region	Sequence	Fluorophore
Telomere	(AATCCC)^3^	Cy3
Centromere of chromosome 2	Dako. The exact sequence is proprietary.	Cy3
Pancentromeric and telomeric FISH		
Target region	Sequence	Fluorophore
Telomere	(AATCCC)^3^	Cy3
Human α-satellite	(AAACACTCTTTTTGTAGA)	FAM

### Assessment of cell viability in GSC

To assess cell viability after RHPS4 exposure, GSCs were mechanically dissociated and plated at a density of 2 × 10^4^ cells/ml in 96-well microtiter plates. After 16 h, RHPS4 was added to the cells. ATP levels were measured at different time points as a surrogate of cell viability using CellTiter-Glo™ (Promega Inc., Madison, WI) following the manufacturer’s instructions. The mean of the raw luminescence values from triplicate wells treated with vehicle alone (mLC), was used as reference to interpolate percent viability from wells treated with drugs (VD), using the following formula [33]:

$$ \mathrm{VD}=\left(\mathrm{LD}/\mathrm{mLC}\right)\times 100 $$

### Flow cytometric analysis

S-phase progression was evaluated in GSC line #1 by BrdU pulse and chase experiments with the aim to understand the progression of cells in S-phase at the time of treatment and their possible delay over time. For this purpose, after treatment cells were pulsed 3 h with 10 μM bromodeoxyuridine (BrdU), then washed and grown in fresh medium and harvested at 4, 8 and 24 h. Each sample was fixed, permeabilized and the histones were dissociated with 2 M HCl as previously described [34]. BrdU-positive cells were detected with an anti-BrdU primary antibody diluted 1:100 (DAKO Cytomation) and with an anti-mouse-Alexa488 conjugated diluted 1:100 (Invitrogen). Both antibodies were incubated for 1 h RT in the dark. All samples were counterstained with propidium iodide (PI; Sigma-Aldrich) for DNA/BrdU biparametric analysis.

### Statistical analysis

Differences per treatment group were calculated using the two-tailed Student’s t-test. Differences of repeated measurements with different assays were calculated with the Mann Whitney test. All statistical tests were performed with GraphPad Prism software (GraphPad, San Diego, CA). Differences with a *P* value of less than .05 were considered statistically significant.

## Results

### RHPS4 and IR combined treatment inhibits the tumor growth and prevents tumor recurrence in vivo

**Fig. 1:**
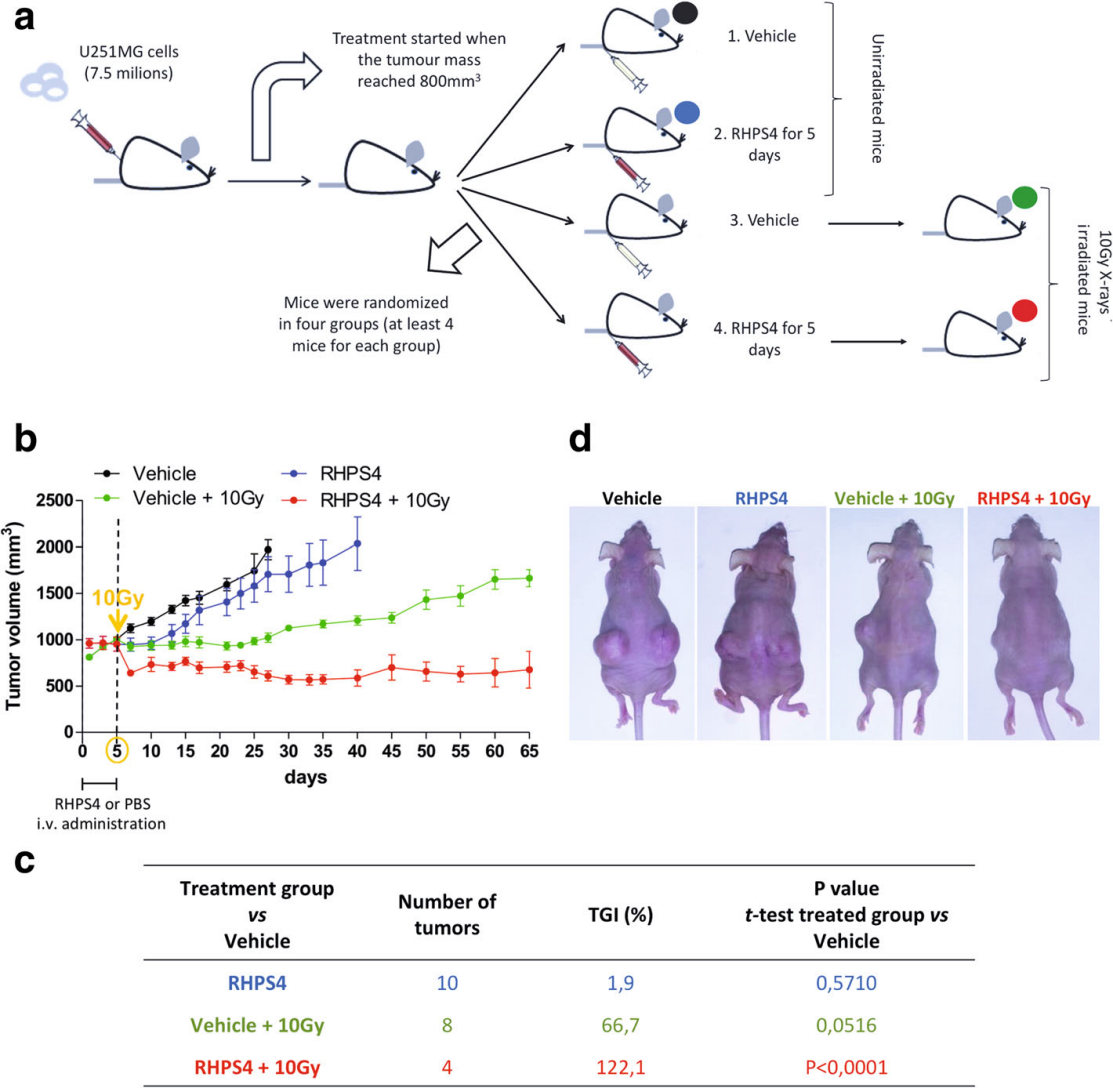
RHPS4 and IR synergize to inhibit the growth of glioblastoma xenograft preventing tumor relapse. Graphical representation of the in vivo experimental plan. U251MG cell xenografted mice were randomized in four groups: Vehicle, RHPS4, Vehicle + 10 Gy and RHPS4 + 10 Gy. RHPS4 (10 mg/kg per day) or PBS (vehicle) were administered through intravenous injection for 5 days, then mice were irradiated with a single dose of 10 Gy of X-rays (**a**). The graph in **b** shows the tumor growth kinetic relative to each treated group started when tumor mass reached 800 mm^3^ in volume. In panel **c** is shown the tumor growth inhibition (TGI%) of treated tumors for each experimental group compared with vehicle group. Representative images of U251MG cell xenografted mice 65 days post-treatment with a clear regression of tumor mass in the combined treatment group (RHPS4 + 10 Gy) (**d**)

Previous in vitro results showed that RHPS4 inhibited cell growth in GBM cell lines and sensitized to IR treatment in a synergistic manner through a telomere-dependent mechanism [17]. To validate the in vivo efficacy of RHPS4 and IR combined treatment, U251MG cells were injected subcutaneously into the flank of CD1 nude female mice. Animals were randomized in four groups as summarized in Fig. 1a. As shown in Fig. 1b and d, tumors in the control group (Vehicle) grew rapidly; after 20 days, in fact, the tumor average size is 2.4-fold greater than the beginning. The growth kinetics of tumors in mice treated with RHPS4 for 5 days was comparable to that observed in the vehicle group, with a final TGI of 1.9% (Fig. 1b-d). In the first 30 days of experiment, irradiation alone (Vehicle + 10 Gy group) significantly inhibited the tumor growth compared with control group; afterward, a slight but constant regrowth of tumor mass was recorded until the end of the experiment (Fig. 1b and d). Nevertheless, the final value of TGI was 66.7%, approaching an acceptable significance level (Fig. 1c; *P* = 0.0516). When mice were first treated with RHPS4 and then irradiated (RHPS4 + 10 Gy, group), a striking block in the tumor growth was observed. At all time-points examined, the tumor dimension was significantly reduced when compared to other groups (Fig. 1b and d), indicating that this combination synergistically inhibited tumor growth in comparison with single treatments (RHPS4 or X-rays alone). Furthermore, the final value of TGI obtained in this group (TGI% = 122.1%) clearly indicate that double treatment caused regression of tumors to far below the starting volume and, importantly, no tumor re-growth was observed during the post-treatment observation period (65 days).

### RHPS4 induces DNA damage in vivo in U251MG-derived tumors

Intravenous administration of RHPS4 was able to reach the site of tumor growth and induce 53BP1 foci formation as shown by immunostaining of tumor sections recovered from mice exposed either to 10 mg/Kg/die RHPS4 for 5 or 10 days and in matched controls (only vehicle). Analysis of either 53BP1 foci/cell or frequency of cells positive to 53BP1 (cells that display > 4 foci per cell) indicated that RHPS4 administration was able to induce DNA damage in vivo in GBM tumor cells (Additional file 1: Figure S1). As previously shown in vitro, DNA damage induced by RHPS4 is mainly, but not exclusively, due to the binding of G4 located in telomeric regions and to their dysfunctionalization [16]. Interestingly, DNA damage levels were similar between mice treated for 5 and 10 days with RHPS4, indicating a non-linear correlation between RHPS4 treatment duration and DNA damage (Additional file 1: Figure S1). 53BP1 foci/cell and positive cells reach a plateau phase before the 5^th^ day of treatment and maintain similar yield of damage until day 10 (Additional file 1: Figure S1). This evidence suggests that cellular sensitivity to RHPS4 is due to the presence of a subset of sensitive telomeres that may be targeted and dysfunctionalized by RHPS4 in the first days of treatment.

### Characterization of stem-like cells derived from U251MG

**Fig. 2:**
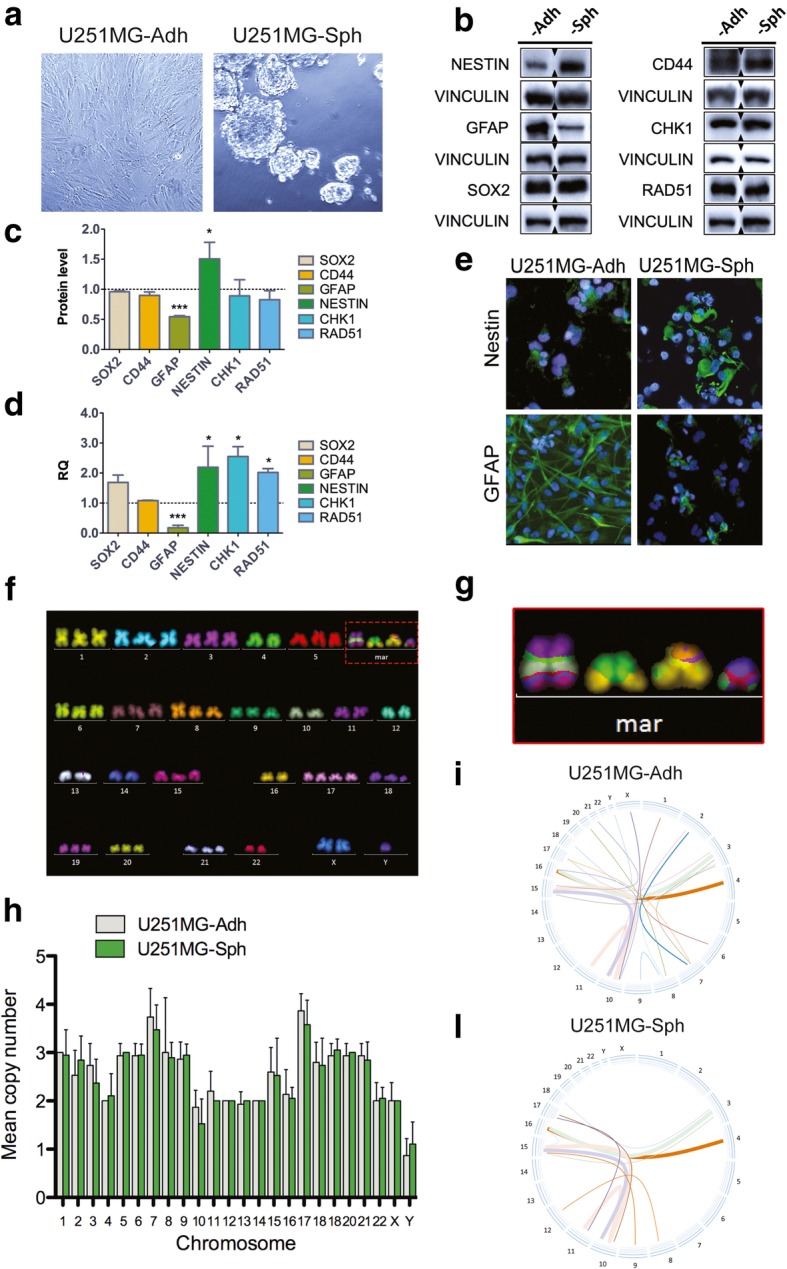
Stem cell markers and cytogenetic characterization of U251MG-Sph. Representative images of adherent U251MG cells and spheres derived from the same cell line (**a**). Western blot of NESTIN, SOX2, CD44 and GFAP in U251MG-Ahd and -Sph cells (**b**). Densitometric analysis revealed a significant reduction of GFAP and a significant increase of NESTIN in U251MG-Sph compared to U251MG-Adh (**c**). Data were also confirmed by qRT-PCR (**d**). Images of immunofluorescence versus NESTIN and GFAP confirmed western blot data (**e**). Most common karyogram observed in U251MG-Adh cells as revealed by mFISH (**f**). Derivative chromosomes are indicated as mar and involved chromosomes 11–10-15, 10–15, 16–4 and 16–3 (**g**). Ploidy of U251MG-Adh and -Sph was completely superimposable (**h**), whereas as shown in circos graphs, frequency of chromosomal exchanges is higher in U251MG-Adh (**i**) than in U251MG-Sph (**l**). * *P* < 0.05, ** *P* < 0.01, *** *P* < 0.001 (Student’s t-test)

To assess whether stem cell compartment was specifically targeted by RHPS4, U251MG stem-like cells (U251MG-Sph) were isolated and growth as suspending spheres enriched with stemness characteristics, from the parental U251MG total cell line (U251MG-Adh) (Fig. 2a). To assure the robustness of U251MG stem-like spheres isolation protocol we firstly determined the immunophenotype of U251MG-Adh and U251MG-Sph cells. Analysis of *CD133*, *CD44*, *SOX2*, *NESTIN* and *GFAP* expression was performed through immunofluorescence, western blotting, and qRT-PCR experiments. Although both cell lines exhibited a lack of immunoreactivity for CD133 (data not shown) as previously reported [35], U251MG-Sph cells showed higher levels of NESTIN at the protein and mRNA levels when compared to U251MG-Adh cells (Fig. 2b, c, d, and e). SOX2 and CD44 levels were comparable in both cell types (Fig. 2b, c, and d). Notably, under the two culture conditions U251MG cells exhibited distinguishing immunoreactivity for GFAP, that is a marker of the differentiated glial cell type. In particular, when compared to U251MG-Sph, U251MG-Adh cells showed significantly higher immunoreactivity and gene expression for GFAP (Fig. 2b, c, d, and e). Moreover, *CHK1* and *RAD51* gene expression and protein levels were analyzed in U251MG-Adh and U251MG-Sph. These proteins are not stem markers but are often upregulated in cancer stem-like cells (CSCs) and in particular in GSCs [36, 37]. Interestingly, we found a two-fold significant overexpression of the two genes whereas protein levels did not change significantly (Fig. 2b, c, and d). In order to evaluate the overall genomic stability, telomere length, telomere fragility, telomerase activity, and both cytogenetic and biochemical analysis were performed in U251MG-Adh and U251MG-SC-Sph cells. Although we did not find differences in cell ploidy (modal number was ~ 65 in both cell lines) (Fig. 2f and h), mFISH staining revealed that chromosomal rearrangements were more frequent in U251MG-Adh than in U251MG-Sph cells (Fig. 2i and l). Indeed, except for four conserved derivative chromosomes that were present in more than 90% of the cell observed (derivative chromosomes are shown as markers (mar) in karyogram Fig. 2f and enlarged in Fig. 2g), U251MG-Adh cells displayed a number of rearrangements significantly higher than those observed in U251MG-Sph as clearly shown in circos graphs (Fig. 2i and l). This data indicates an enhanced control of genomic stability and was in accordance with the net gain of new chromosomal aberrations detected comparing non-stem and stem tumor cells derived from high-grade gliomas and medulloblastomas [38]. The lower chromosomal instability of the stem-like population may be ascribed to fast and efficient DNA repair mechanisms evolved in stem and progenitor cells, whereas, upon differentiation, a certain degree of somatic mutations becomes more acceptable and, consequently, DNA repair dims [39].

Analysis of telomere length and telomerase activity showed that telomere metabolism was differently regulated in U251MG-Adh and U251MG-Sph. Telomere length measurement showed significantly longer telomeres in stem-like cells when compared to their differentiated counterpart (9.1 and 5.6 T/C%, respectively) (Additional file 2: Figure S2 A, B, and C). Longer telomeres in -Sph cells were also coupled with a higher telomere fragility as demonstrated by the higher frequency of telomere doublets per chromosome (Additional file 2: Figure S2 D) in accordance with the higher basal replicative stress (RS) reported in GSCs [36, 37].

The two cell types displayed also different levels of telomerase activity (TA); in detail U251MG-Adh cells showed a two-fold higher TA than U251MG-Sph (Additional file 2: Figure S2 E). Lower telomerase activity was also previously reported comparing neural cancer cells and CSC [40]. Overall, the analysis of telomere *status* revealed significant differences between glioblastoma derived stem-like cells and the whole adherent cell line.

### RHPS4 inhibits cell proliferation in U251MG-derived neurospheres and in GSCs from patients irrespectively from IR exposure

**Fig. 3:**
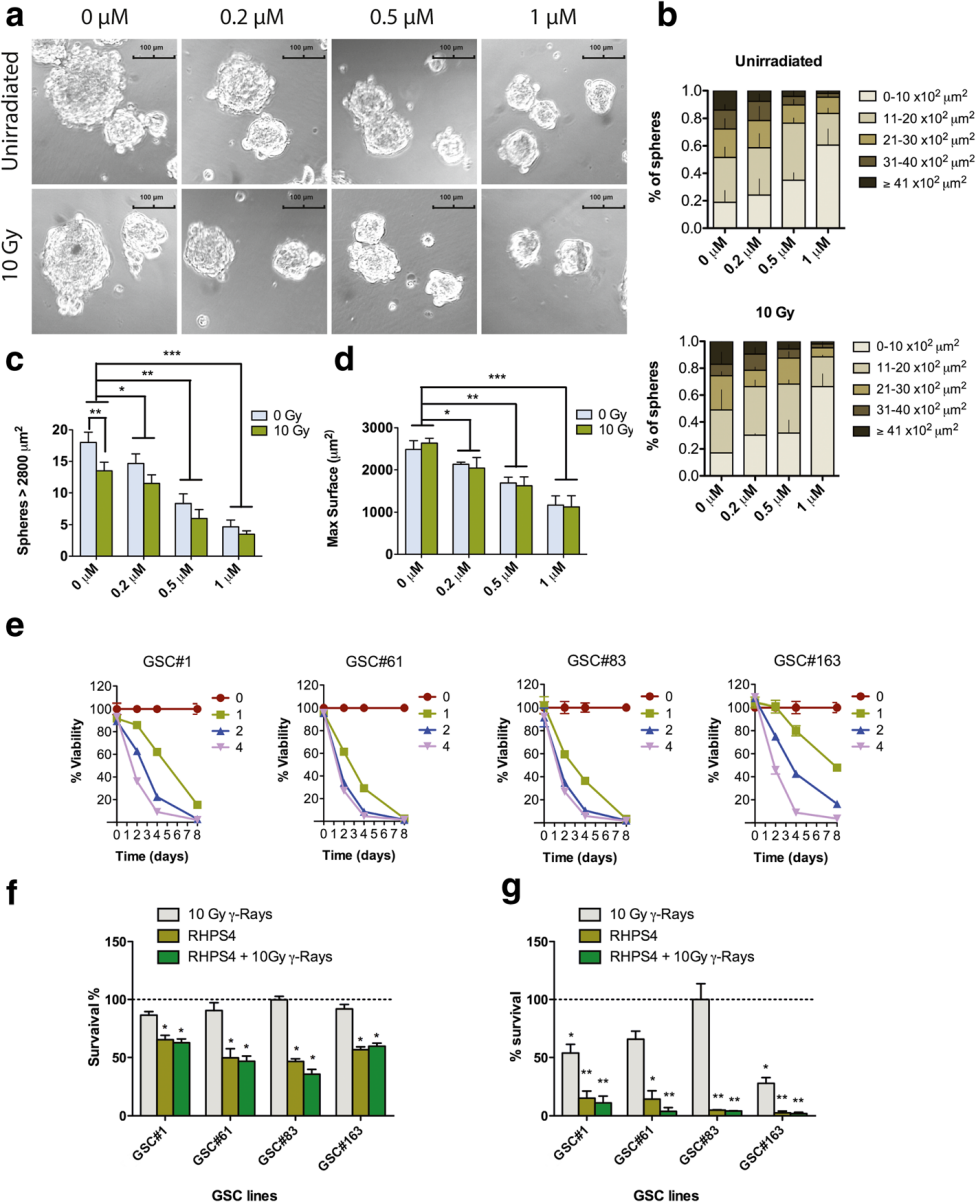
Cytotoxic effect of RHPS4 in single treatment and in combination with IR. Images of U251MG-derived neurospheres treated with increasing concentrations of RHPS4 (0.2–1 μM for 5 days) and then exposed to 10 Gy of X-rays (**a**). Neurospheres maximal surface was automatically calculated by IS-Capture software after manual surrounding of each sphere. Maximal surface data were grouped in 5 different classes (0–10, 11–20, 21–30, 31–40 and ≥ 41*10^2^ μm^2^) for both unirradiated and 10 Gy-exposed U251MH-Sph cells. Data have been reported as percentage of the total number of spheres analyzed and represent mean values ± s.d. (*n* = 3) (**b**). Spheres number and max surface in samples exposed to RHPS4 and IR were shown in (**c**) and (**d**), respectively. Data represent mean values ± s.d. (*n* = 3). Growth curves of GSCs from patients treated with RHPS4 (1, 2, 3 and 4 μM) and followed for 8 days (**e**). Data represent mean values ± s.d. (*n* = 2). Effect of RHPS4 and γ-rays combined treatment on cell growth in GSCs from patients. Cell growth was evaluated after 3 (**f**) and 7 days (**g**) from irradiation. Combined treatment was performed treating cells with IC_25_ calculated at 4 days and then exposing them to 10 Gy γ-rays. Data represent mean values ± s.d. (*n* = 2). * *P* < 0.05, ** *P* < 0.01, *** *P* < 0.001 (Student’s t-test)

Our data showed that RHPS4 is a very effective inhibitor of cell proliferation in both GSC models used. Data from the neurospheres assay showed that after 10 days from treatment, RHPS4 was able to reduce in a linear dose-dependent manner (R^2^ = 0.93) both number and size of spheres (Fig. 3a, b, c, and d), with the maximum effect observed at the concentration of 1 μM (Fig. 3b, c, and d) where we found a 60% reduction in spheres number and about 70% reduction in spheres size (Fig. 3c, and d). Surprisingly, no radiosensitization was observed when samples were exposed to 10 Gy of X-rays (Fig. 3b, c, and d). Accordingly with these data, RHPS4 was also able to drastically reduce cell proliferation in vitro in four different patient-derived GSC lines. Although confirming that GSCs are more resistant to drug treatments compared to differentiated cancer cells [7, 41], in all of the GSC lines analyzed RHPS4 inhibited cell growth in a dose- and time-dependent manner (Fig. 3e). The IC_25_ values calculated after 4 days of treatment were: 0.7 μM for GSC #1; 0.8 μM for GSC #61; 0.5 μM for GSC #83; 1.2 μM for GSC #163 whereas IC_25_ for U251MG was 0.16 μM. Remarkably longer treatments (7 days) drastically reduce cell proliferation in patient-derived GSCs. Based on these results, we investigated whether RHPS4 exposure could enhance GSCs sensitivity to IR. Therefore, GSCs were treated with RHPS4 IC_25_ for 4 days and then exposed to single dose of γ-rays (10 Gy), selected as the closest to the maximum tolerated dose for adult brain and optic pathways on unfractionated radiosurgery [42]. Evaluation of cell viability (3 and 7 days) after irradiation indicated that GSCs sensitivity to IR was not improved in combined treated samples (Fig. 3f and g).

### RHPS4 induces telomere-mediated chromosomal aberrations in differentiated U251MG cells but not in U251MG-derived neurospheres and in patient-derived GSCs

**Fig. 4:**
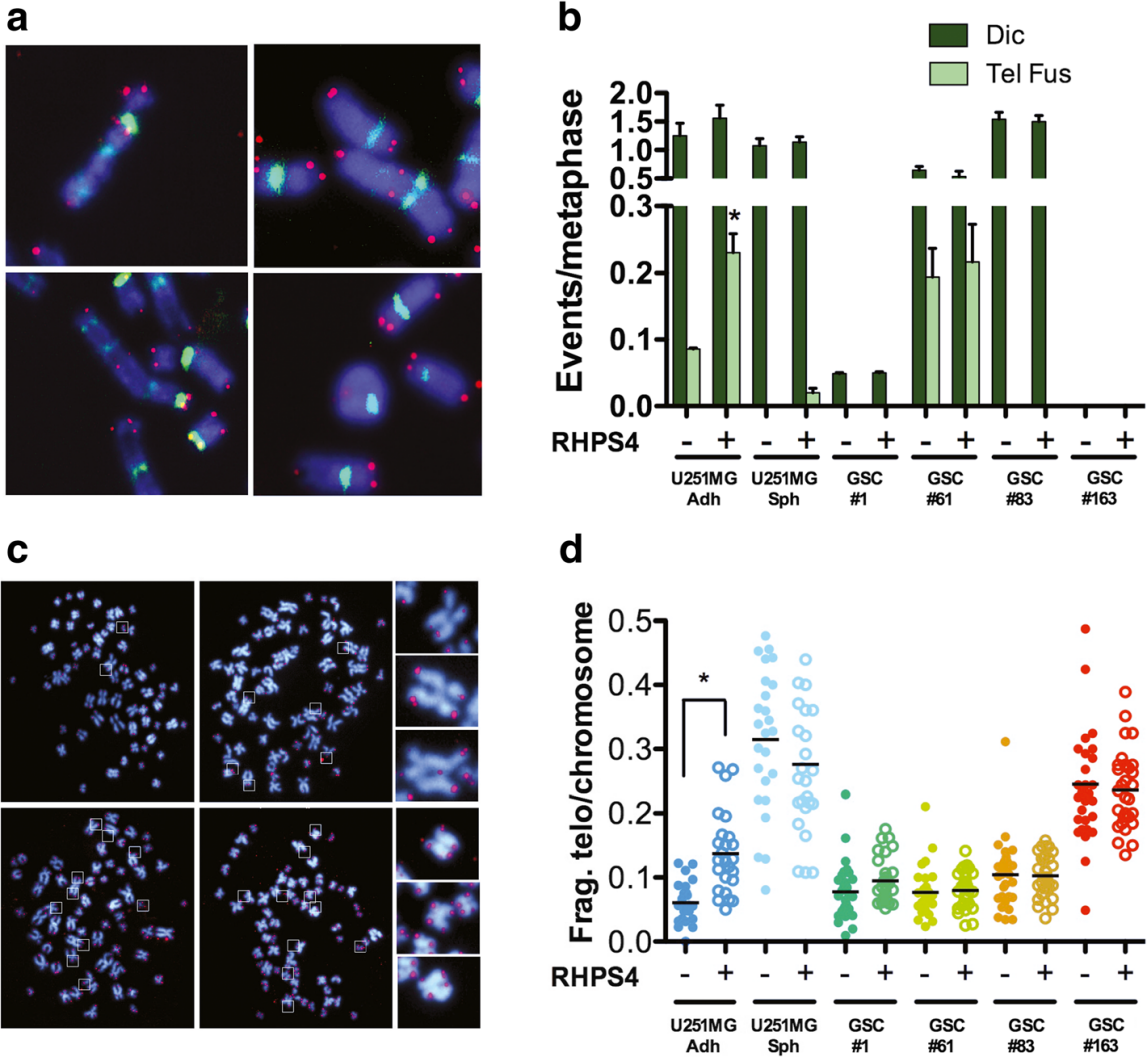
RHPS4 induces telomeric fusions and telomeric fragility in glioblastoma differentiated cells but not in GSCs. Representative images of telomere fusions involved in the formation of dicentric, tricentric and ring chromosomes observed in the U251MG-Adh cell line treated with 0.5 μM RHPS4 for 5 days (**a**). Frequency of classical dicentrics (dic) and dicentrics generated from telomere fusions (tel fus) in the U251MG derived cell lines and in GSCs lines from patients (**b**). Data represent mean values ± s.d. (*n* = 2). Representative images of U251MG-Adh cells in which are present several fragile telomeres (surrounded by boxes). Some of them were enlarged on the right side of the Fig. (**c**). Frequency of fragile telomeres per chromosome in the U251MG derived cell lines and in GSCs lines obtained from patients treated or not with RHPS4. Data represent the frequency of each metaphase scored and black bars denotes mean values (**d**). (*n* = 2). * *P* < 0.05 (Student’s *t*-test)

RHPS4 radiosensitization of GBM cells is mainly driven by telomere dysfunction [16]. In order to understand whether the lack of radiosensitization observed in stem cells may be ascribed to a telomeric resistance to RHPS4, we performed a telomeric analysis in order to evaluate telomere-mediated chromosomal aberrations and/or telomere length modulation in both U251MG-Sph and patient derived-GSCs. Results indicated that, differently from U251MG-Adh, both the stem cell models did not respond to RHSP4 at telomeric level and did not show any induction of dicentric chromosomes or telomeric fusions suggesting the capacity to bypass stabilized G4 structures at telomeres (Fig. 4a and b). In agreement with the high genetic and karyotypic complexity of GBM cells, we found near-to-tetraploid modal number in three out of four patient derived cell lines; moreover, we observed the clonal presence of dicentric chromosomes in lines #1, #61, and #83, and the presence of telomeric fusions in line #61 (Additional file 3: Figure S3A).

RHPS4 induces telomere doublets that are double or discontinuous telomere signals at the chromatid ends [43]. In some cases, the multiple signals were spatially separated from the chromatid terminus, as if the telomeric DNA has failed to condense or was broken [44]. We refer to these various abnormal telomeric patterns as fragile telomeres (Fig. 4c). Analysis of fragile telomeres confirmed the different telomeric response to RHPS4 of differentiated compared to cancer stem cells. In particular, we found a significant induction of fragile telomeres in U251MG-Adh cells with frequencies two-fold higher in RHPS4 treated cells than in untreated controls (Fig. 4d). On the other hand, U251MG-SC-Sph and GSCs from patients did not display any increase in telomeric fragility confirming the lack of telomeric effect of the ligand in GSCs (Fig. 4d). Moreover, analysis of telomere lengths, using centromere-calibrated QFISH [32, 45], revealed a very heterogeneous telomere length ranging from 4 to 15 T/C% (as reference normal primary fibroblasts HFFF2 at passage 25 have a telomere length of 14 T/C%). In particular, as expected for short treatment duration (5 days) [16], the analyzed cell lines showed neither mean telomere length modulation (Additional file 3: Figure S3B) nor enrichment of the shortest telomere fraction (Additional file 3: Figure S3C).

### RHPS4 mediates the reduction of RAD51 and CHK1 in differentiated and stem-like cancer cells

The absence of telomere-involving chromosomal aberrations led us to investigate additional RHPS4 targets to explain the potent proliferation inhibition observed in cancer stem cells. Due to the ability of G4 ligands to induce replicative stress and DNA damage, we analyzed a panel of proteins involved in DNA damage signaling, repair, and checkpoint activation (i.e., ATM, pATM, ATR, pATR, CHK1, pCHK1, CHK2, pCHK2, RAD51, PCNA, Ku80, DNAPK).

**Fig. 5:**
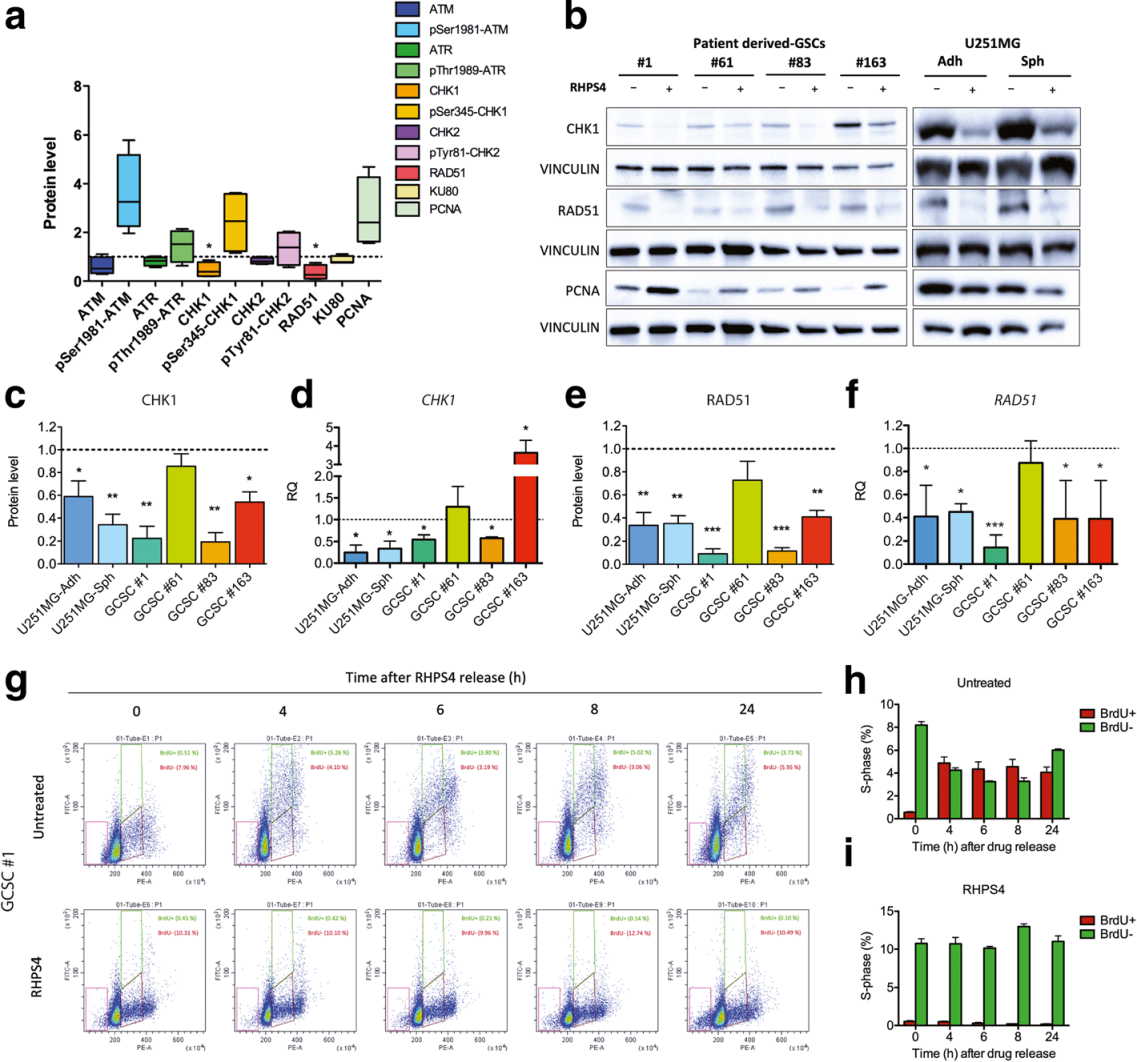
RHPS4 induces reduction of CHK1 and RAD51 and determine S-phase blockage in GSCs. Analysis of proteins involved in DNA damage response and checkpoint activation in patient derived-GSCs (**a**). Representative blots for CHK1, RAD51 and PCNA in patient derived-GSCs and U251MG (−Adh and -Sph) (**b**). CHK1 and RAD51 protein level and gene expression in all the cell lines analyzed (**c**, **d**, **e**, **f**). Data represent mean ± s.d. (*n* = 3). * *P* < 0.05, ** *P* < 0.01, *** *P* < 0.001 (Student’s t-test). Assessment of the BrdU-incorporating cells in samples exposed for 4 days to RHPS4. Cells were pulsed for 3 h with BrdU and, after RHPS4 release, cells were chased for additional 24 h (cell were fixed after 4, 6, 8 and 24 h) (**g**). Quantification of BrdU positive (BrdU+) and negative (BrdU-) cells in untreated (**h**) and RHPS4-reated cells (**i**). Note the total depletion of BrdU+ observed over-time in RHPS4 treated samples

Our data highlighted that RHPS4 activated the DNA damage response through both ATM and ATR kinases, which resulted phosphorylated at Ser1981 and Thr1989, respectively (Fig. 5a). In particular, we observed that RHPS4 caused the activation of the ATR-CHK1 pathway (Fig. 5a) as shown by the phosphorylation level of ATR and CHK1 observed in almost all the patient derived GSC lines. As previously observed by our group in U251MG-Adh [46], RHPS4 was able to induce CHK1 phosphorylation concomitantly reducing also the basal level of total CHK1 (Fig. 5b). Overlapping data were also obtained on GSC #1, #83, #163 and U251MG-Sph (Fig. 5c). At the mRNA levels, we observed that *CHK1* was downregulated in U251MG-Adh and U251MG-Sph, in GSC line #1 and #83 but not in line #163 (the latter significantly upregulated). These data indicate that, in addition to transcriptional mechanisms, also post-transcriptional and post-translational regulation may be involved in protein levels reduction (Fig. 5d). In contrast with the other cell lines, GSC line #61 modulates neither *CHK1* expression nor its protein level in response to RHPS4 (Fig. 5c and d).

Accordingly with data showing that the highest level of CHK1 expression was coupled with the highest resistance to RHPS4, we decided to investigate if CHK1 depletion by three different lentiviral shRNA (shCHK1 B5, E1 and F11) could enhance the sensitivity to RHPS4 in in GSC#163. Evaluation of CHK1 expression at both protein and mRNA level confirmed the silencing of CHK1, indicating a 20–30% of residual protein compared to the non-targeting shRNA control (NTC) (Additional file 4: Figure S4 A-C). Treatment of all shCHK1 cell lines with RHPS4 showed a significant increase of sensitivity at the lowest concentration tested (1 μM) (Additional file 4: Figure S4 D-E). Analyzing the basal level of total CHK1 in GSC#163 after RHPS4 treatment, we observed a reduction of protein levels in a dose-dependent manner (Additional file 4: Figure S4 F-G). These data demonstrate that RHPS4 treatment at higher doses induces a strong reduction of CHK1 expression that nullify the effect of the silencing, confirming the involvement of CHK1 in GSC growth inhibition mediated by RHPS4.

Remarkably, also RAD51 protein levels were strongly reduced in response to RHPS4 treatment. In particular, we observed a 60 to 90% reduction of RAD51 levels in U251MG-Adh, U251MG-Sph, GSC#1, #83, and #163 treated cells compared to untreated ones; no significant reduction was observed in line #61 (Fig. 5e). Expression profile of *RAD51* transcript levels demonstrated that the protein reduction was determined by a reduced gene expression (Fig. 5f), thus indicating *RAD51* as a novel putative RHPS4 target-gene. As RAD51 and CHK1 are modulated in a cell cycle-dependent manner (i.e., highly expressed in S and G2 phase), we checked for the expression levels of the S-phase specific protein PCNA to exclude that the RHPS4-dependent downregulation of RAD51 and CHK1 proteins was due to cells accumulation in G1 phase. Results obtained showed an increase of PCNA levels in all the RHPS4-treated GSCs, thus indicating a blockage in the S-phase and excluding that *CHK1* and *RAD51* reduction was cell cycle-dependent (Fig. 5b).

To further confirm this result, the patient-derived GSC line #1 was tested for BrdU incorporation after RHPS4 treatment. Pulse and chase BrdU incorporation experiments showed that untreated glioma cells are characterized by a very slow S-phase progression as expected for cells with stemness characteristics. Eight hours after BrdU removal, most of the BrdU positive (BrdU+) cells were located at S/G2 phases, whereas after 24 h treatment a BrdU+ population that have passed mitosis appeared in G1 phase. Conversely, in RHPS4-treated cells we failed to observe either an S-phase progression or a BrdU incorporation (green square gate). Furthermore, an evident sub-diploid peak indicating cell death induction appeared after RHPS4 treatment (red square gate in Fig. 5g). In addition, a prolonged BrdU incorporation time (24 h) indicated that RHPS4-treated cells were quiescent with almost total abrogation of cycling cells. GSC lines #61, #83, and #163 were not analysed for this endpoint as they failed to incorporate BrdU also after longer pulses (up to 6 h). These data could be explained by the very long doubling times of GSC lines (48–96 h).

Beside the analysis of DNA damage signaling and checkpoint activation, we also looked at the stemness markers *SOX2*, *NESTIN*, and *CD44* in response to RHPS4 treatment. Overall, no significant modulation of both protein levels and gene expression was observed for any of the markers analyzed (Additional file 5: Figure S5).

## Discussion

The telomeric targeting as a means to sensitize cancer to DNA-damaging cytotoxic treatments (including radiotherapy) has become of increasing interest with the availability of new telomere targeting agents such as telomeric G4 ligands. The telomeric G4 ligand RHPS4 is one of the most effective and well-studied G4-stabilising molecules [20]. It causes telomere deprotection and inhibition of cell proliferation in several types of cancer cells [22, 23] and is also a potent radiosensitizers as shown in vitro on glioma cell lines when combined to either X-rays or therapeutic carbon ions beam [16, 17]. Here, we show that RHPS4 maintains its radiosensitizing ability also in vivo in a U251MG heterotopic xenograft mouse model. In particular, data showed that, unlike mice exposed to single agent, combo treated mice showed a very potent and durable inhibition of the tumor growth as observed until the 65th day after treatment. Notably, differently from most of the other studies, we started RHPS4 and IR combined treatment on mice harboring aggressive and fast growing tumor mass mimicking the therapeutic treatment of well-rooted tumors. Remarkably, the absence of tumor relapse in combo treated mice let us to hypothesize the targeting of GSCs as a possible therapeutic strategy and prompted us to explore this option in vitro. To dissect the response of GSCs to RHPS4 and IR in single and combined treatment, two models were used: (*i*) U251MG stem-like component (U251MG-sph) isolated from the U251MG total cell line (U251MG-Adh), and (*ii*) 4 well-characterized primary GSCs obtained from GBM patients (WHO grade IV) [25, 26].

Following a detailed molecular and cytogenetic characterization of the U251MG derived stem-like model (see Fig. 2 and Additional file 2: Figure S2), we performed experiments aimed at determining the sensitivity to RHPS4 and IR in single or combo treatments. Data indicated that U251MG-Adh and -Sph sensitivity to RHPS4 was similar (about 0.5 μM for both) whereas spheres were very resistant to combined treatment. Indeed, only a 25% reduction in spheres number was observed in irradiated samples compared to control, without any difference in spheres size. However, conversely to data obtained in U251MG -Adh cells [16], RHPS4 failed to radiosensitize stem-like cells.

To further confirm data observed in stem-like cells, experiments were carried out also in GSCs obtained from GBM patients [25, 26]. Accordingly to data indicating high drug resistance [47], GSCs displayed a higher resistance to RHPS4 compared to U251MG-Sph cells, with IC_25_ values ranging from 0.5 to 1.2 μM (i.e. 0.7, 0.8, 0.5 and 1.2 for GSC#1, #61, #83 and #163, respectively) as evaluated after 4 days. However, longer treatment (7 days) determined a massive cell death, with a reduction of IC_25_ to 0.07, 0.05, 0.04 and 0.37 μM for the cell line #1, #61, #83, and #163, respectively, pointing to a very potent effect of RHSP4 as single agent. Interestingly, the most radioresistant and TMZ resistant cell line (i.e., GSC #61) [48] appeared the most sensitive to RHPS4, whereas the most radiosensitive and TMZ sensitive cell line (i.e., GSC #163) [48] resulted the most resistant to RHPS4. This evidence suggests that different pathways are involved in the response to IR and RHPS4, supporting the notion that RHPS4- and IR-combined approach may represent a very promising strategy in GBM treatment.

Despite the high sensitivity of GSCs to RHPS4 and in agreement to what observed in stem-like derived U251MG, we failed to observe any radiosensitizing effect of RHPS4. As previously demonstrated by our lab, one of the mechanisms behind the RHPS4 radiosensitizing properties is the induction of telomere damage and hence lethal chromosome aberrations such as telomere fusions [16, 17]. Interestingly, although the ability of RHPS4 to induce telomere fusions was documented in different cell lines including U251MG-Adh [16, 43], such kind of aberrations were not detected in U251MG-Sph cells and GSCs. It is well known that the G4-ligand-induced DNA damage response at telomere depends on replication stress (RS), due to the physical impediment to DNA polymerase posed by stabilized G4 [49]. The so-called fragile telomeric sites or telomeric doublets represent a well-accepted marker of RS at telomeres [44]. In this context, RHPS4 determined a significant increase in fragile telomeres frequency in U251MG-Adh cells confirming data reported in literature [43], whereas no differences were found when comparing RHPS4 treated and untreated U251MG-Sph and GSCs. Taken together these data point to a higher telomeric resistance of tumor stem-like to RHPS4 that ultimately reduce also the radiosensitizing properties of the G4 ligand. This led us to speculate that other non-telomeric targets in GSCs might be responsible for the extensive inhibition of cell proliferation observed both in vivo and in vitro. With the aim of finding alternative targets of RHPS4 outside the telomere, we analysed the effect of this molecule on the expression of a panel of proteins involved in DSBs repair and RS. We found that RHPS4 markedly reduced the level of RAD51 and CHK1 in U251MG-Adh, −Sph and GSCs. Notably, both *RAD51* and *CHK1* genes displayed in their promoter G4 putative binding sites or gene bodies with G-scores higher than 37, that is a value very close to that reported for telomeres (QGRS database telomeric G-score: 42). This suggests that RAD51 and CHK1 may represent possible novel RHPS4 target genes. Notably, depletion of RAD51 and CHK1 has been proposed as a strategy to radiosensitize GSCs. Indeed, targeting of CHK1 (and CHK2) in GSCs abrogates G2-M checkpoint function and increases radiosensitivity [7, 33, 37, 50], whereas RAD51 depletion results in highly radiosensitized GSCs [6, 12]. Overall, these data seem to be in contrast with our results showing the lack of RHPS4-dependent radiosensitizing effects in tumor stem-like cells. However, our hypothesis is that the mechanism by which RHPS4 reduces proliferation in GSC is linked to RAD51 and CHK1 reduction also in the absence of radiosensitization. Indeed, it is well known that RHPS4 determines RS through the stabilization of G4 located at telomeres [43], although RHPS4 is also expected to bind a number of non-telomeric G4 in different G-rich genomic regions [21, 51] hampering the RS, constitutively present in GSCs [52]. Our previously data [46] and evidence from the present work indicate that, though CHK1 was normally phosphorylated after RHPS4 treatment, the level of total CHK1 was significantly lowered by RHPS4 treatment. We believe that the downregulation of CHK1, despite its proficient phosphorylation, determines a deficient RS response that increases the yield of replication fork stall in regions harboring stabilized G4. Silencing of CHK1 in GSC #163 further confirmed our hypothesis, indicating that sensitivity to RHPS4 in GSC is strongly affected by CHK1 levels. Stalled replication forks are processed and stabilized by replication fork reversal, a process also known as fork regression [37]. Although the molecular mechanism was not hitherto fully elucidated, fork reversal is prompted by the activity of the HR protein RAD51 [53]. The most accepted mechanism indicates that Mus81 endonuclease cleaves DNA at stalled fork and determines the formation of a one-ended DSB that in turn activates RAD51-mediated recombination [54]. However, in GSCs, in which HR plays a central role in the repair of DSBs [55], the concomitant RHPS4-induced depletion of RAD51 and CHK1 determined the failure in reversal of the stalled replication fork leading, in turn, to collapse and DSB induction also in the absence of IR exposure.

BrdU incorporation analysis in GSCs line #1 sustains this hypothesis, showing that RHPS4 not only determines a strong arrest of cells in the early S-phase, but is also accompanied by the presence of a growing sub-G1 population suggestive of cell death. In agreement with our hypothesis, the sensitivity of GSCs to RHPS4 was proportional to the extent of RAD51 and CHK1 protein level reduction for 3 out of 4 lines analyzed (line #61 excluded). The different response to RHPS4 observed in line #61 it is not surprising as GSCs lines usually display very different metabolic profiles that makes the treatment strategy very difficult to be designed. In turn, this may account for a patient-dependent drug response [56].

## Conclusions

**Fig. 6:**
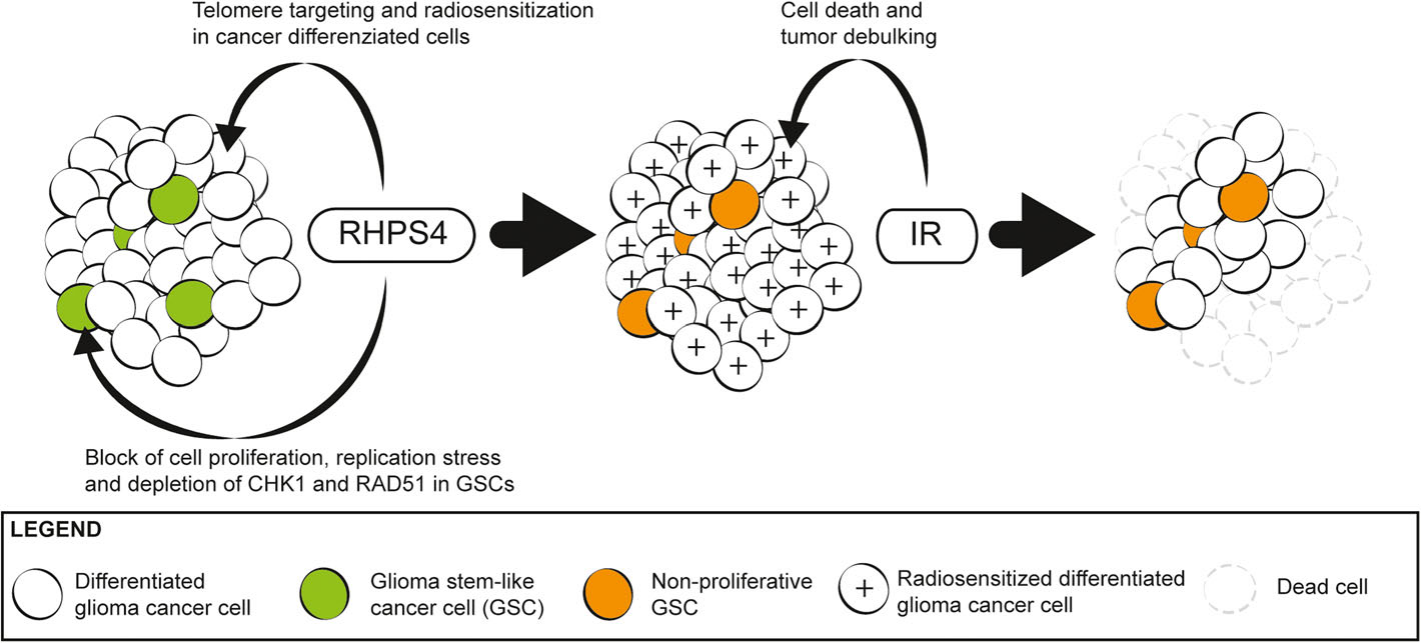
RHPS4 inhibits glioblastoma cell proliferation through a differential targeting of bulky differentiated- and cancer stem-like cells. RHPS4 differently targets differentiated and GSCs cells. Differentiated glioblastoma cells (white circles) are radiosensitized (white circles marked with plus symbol) through the induction of telomere damage and dysfunctionalization [16, 17]. On the other hand, in GSCs (green circles) RHPS4 strongly reduces CHK1 and RAD51 protein levels leading to S-phase blockage, inhibition of cell proliferation (orange circles) and very likely to cell death. The subsequent exposure to IR determines the cell killing of differentiated (radiosensitized) cells and contributes to the tumor debulking. This model fits very well with data obtained in vivo, indicating that RHPS4 and IR combined treatment avoids tumor relapse and reduce tumor mass also in case of full blown and well-rooted tumors

Overall, in the present work we provide evidence that RHPS4 maintains its ability to radiosensitize glioblastoma cells also in vivo, preventing tumor recurrence in mice. The model that we propose is based on a differential mode of action of the G4-ligand in glioblastoma differentiated and stem-like cells. Indeed, while the former are sensitized to IR by the targeting and dysfunctionalization of telomeres through the stabilization of the principal RHPS4 target (i.e., telomeric G4) [16, 17], the latter shows a high sensitivity to the drug in single treatment coupled with the lack of telomeric damage and radiosensitization. We believe that the potent antiproliferative effect of RHPS4 in GSCs is achieved by the induction of RS and by the concomitant depletion of CHK1 and RAD51 that, in turn, lead to DNA damage and cell death (Fig. 6). Moreover, our data confirm that the combined inhibition of cell-cycle checkpoints and DNA repair targets provides the most effective means to overcome resistance of GSC to genotoxic insults.

## Additional files

Additional file 1: **Figure S1.** DNA damage induction observed in U251MG-derived tumors after RHPS4 in vivo treatment. Graphical representation of the experimental procedure. (A) Immunohistochemical analysis of the neural marker GFAP in a tumor section recovered from the flank of an U251MG injected mouse. (B) Immunostaining of the DNA damage marker 53BP1 in tumor sections recovered from mice exposed 10 mg/Kg/die RHPS4 for either 5 or 10 days and in matched controls (only vehicle). (C) Analysis of either 53BP1 foci/cell or frequency of cells positive to 53BP1 (cells that display > 4 foci per cell). (D) Black bars denotes s.d. (4 animals analyzed for each treatment condition). * *P* < 0.05; ** *P* < 0.01 (Student’s t-test). (TIF 18887 kb)

Additional file 2: **Figure S2.** Analysis of the telomere status in U251MG-Adh and -Sph cells. Telomere lengths were analyzed using centromere-calibrated QFISH. Representative images of metaphase spreads from U251MG-Adh and -Sph cells. (A) Distributions of telomere length in U251MG-Adh and U251MG-Sph (B and C). Box plot in C reports means, quartiles and s.d. (*n* = 2). Fragile telomere frequency in U251MG-Adh and U251MG-Sph. Box plot reports means, quartiles and s.d. (*n* = 2) (D). Telomerase activity (TA) in both parental and stem-like derived cells. Data represent mean values ± s.d. (*n* = 2) (E). ** P < 0.01, *** *P* < 0.001 (Student’s t-test). (TIF 17312 kb)

Additional file 3: **Figure S3.** Telomere length analysis in cancer stem-like cells treated with RHPS4. Telomere lengths were analyzed using centromere-calibrated QFISH. Representative images of metaphase spreads from U251MG-Adh and -Sph cells (A). Box Plot of telomere lengths in untreated and RHPS4 treated U251MG-Adh, U251MG-Sph, GSCs#1, #61, #83 and #163. Box plot represents means and quartiles, whiskers represent s.d. and points represent outliers. (B) Percentage of telomeres shorter than 5 T/C% (red bars), comprised between 6 and 10 T/C% (green bars) and longer than 11 T/C% (blue bars) as evaluated in untreated and RHPS4 treated cells (C). (TIF 30408 kb)

Additional file 4: **Figure S4.** Silencing of CHK1 increases GSC response to low concentrations of RHPS4. Protein levels of GSC #163 stably expressing either a non-targeting control shRNA (NTC) or three different shRNAs targeting human CHK1 (named shCHK1 B5, E1 and F11) are shown (A). Densitometric analysis confirmed the significant reduction of CHK1 protein levels in shCHK1 cell lines (B) and a similar reduction was also observed by means of qRT-PCR (C). Growth curves showing the effect of RHPS4 treatment (1, 2, and 4 μM) in both NTC and shCHK1 cells evaluated up to 7 days (D). Cell viability at day 7 from treatment suggests a significant impairment of cell growth in all shCHK1 cells only after 1 μM RHPS4 (E). Conversely, higher concentrations (2 and 4 μM) do not affect cell viability (E). Indeed, RHPS4 treatment in the GSC #163 is able per se to downregulate the levels of CHK1 in a dose-dependent manner (F and G) masking the difference in cell viability between NTC and shCHK1 cells after higher RHPS4 concentrations (2 and 4 μM). Data represent mean values ± s.d. (*n* = 2). * *P* < 0.05, ** *P* < 0.01, *** *P* < 0.001 (Student’s t-test). (TIF 955 kb)

Additional file 5: **Figure S5.** RHPS4 does not affect stem cell markers in U251MG-Sph and GSCs. Western blot analysis of stemness markers SOX2, NESTIN and CD44 in response to RHPS4 treatment in GSCs#1, #61, #83 and #163 (A) and U251MG-Adh and U251MG-Sph cells (B). Protein levels of CD44 (C), SOX2 (D) and NESTIN (E). Box plot represents mean, maximum and minimum values (*n* = 2). Gene expression profile for *CD44* (F), *SOX2* (G) and *NESTIN* (H). Box plot represents mean, maximum and minimum values (*n* = 2). (TIF 21948 kb)

## Data Availability

All data generated or analyzed during this study are included in this published article and its supplementary information files.

## Abbreviations

CSC: Cancer Stem-like Cell
DSB: Double Strand Break
G4: G-Quadruplex
GBM: Glioblastoma Multiforme
GIC: Glioma Initiating cell
GSC: Glioma Stem-like Cell
HR: Homologous Recombination
IR: Ionizing Radiations
NHEJ: Non-Homologous End Joining
RS: Replicative Stress
TGI: Tumor Growth Inhibition
WHO: World Health Organization
