# Supplementary figures and images for: G-quadruplex ligand RHPS4 radiosensitizes glioblastoma xenograft in vivo through a differential targeting of bulky differentiated- and stem-cancer cells
Source: J Exp Clin Cancer Res. 2019 Jul 16;38:311. doi: 10.1186/s13046-019-1293-x (PMC6636127; doi:10.1186/s13046-019-1293-x)

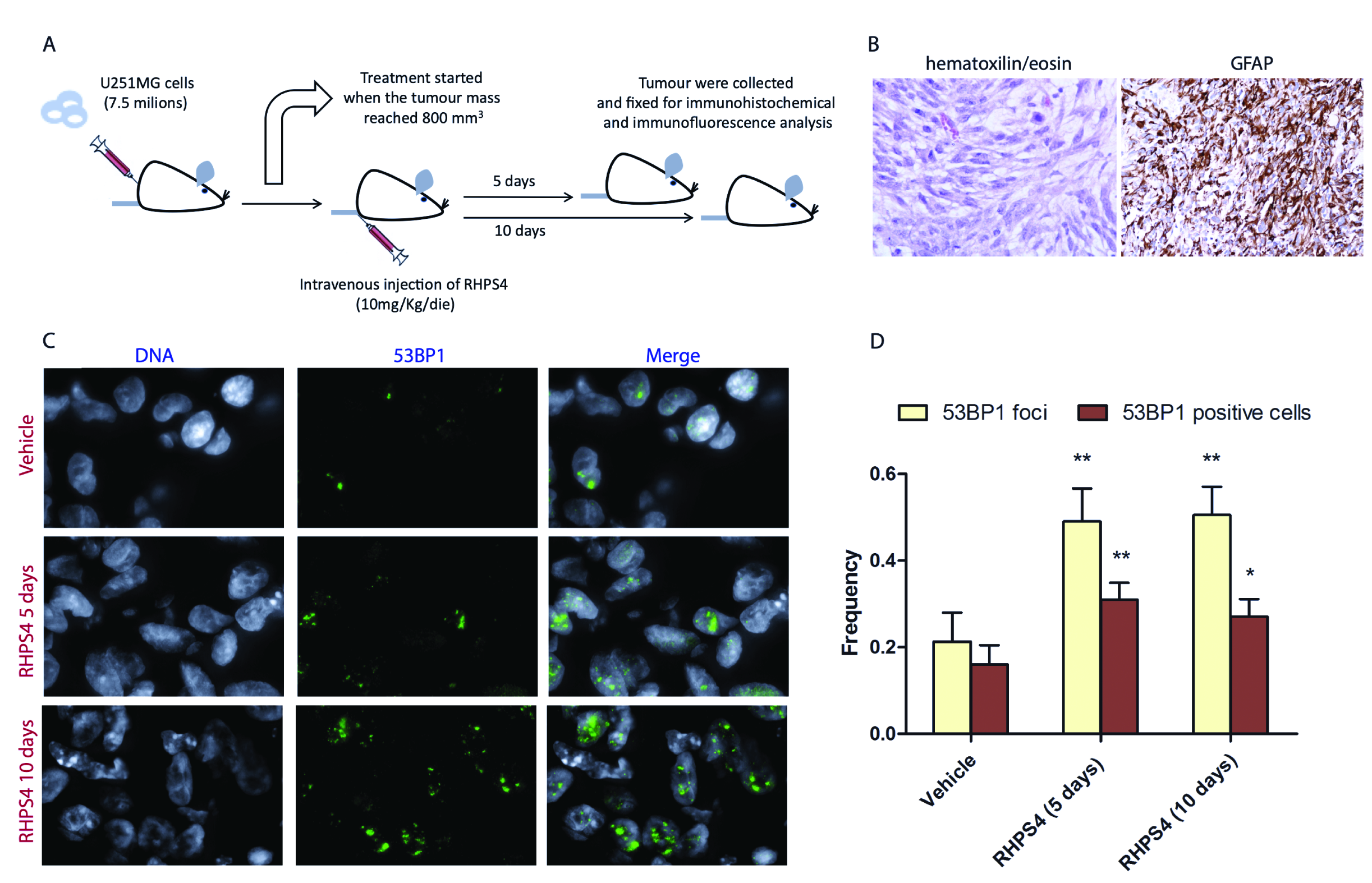

Supplement: Supplementary file 1 — Figure S1. DNA damage induction observed in U251MG-derived tumors after RHPS4 in vivo treatment. Graphical representation of the experimental procedure. (A) Immunohistochemical analysis of the neural marker GFAP in a tumor section recovered from the flank of an U251MG injected mouse. (B) Immunostaining of the DNA damage marker 53BP1 in tumor sections recovered from mice exposed 10 mg/Kg/die RHPS4 for either 5 or 10 days and in matched controls (only vehicle). (C) Analysis of either 53BP1 foci/cell or frequency of cells positive to 53BP1 (cells that display > 4 foci per cell). (D) Black bars denotes s.d. (4 animals analyzed for each treatment condition). * P < 0.05; ** P < 0.01 (Student’s t-test). (TIF 18887 kb) [file 13046_2019_1293_MOESM1_ESM.tif]

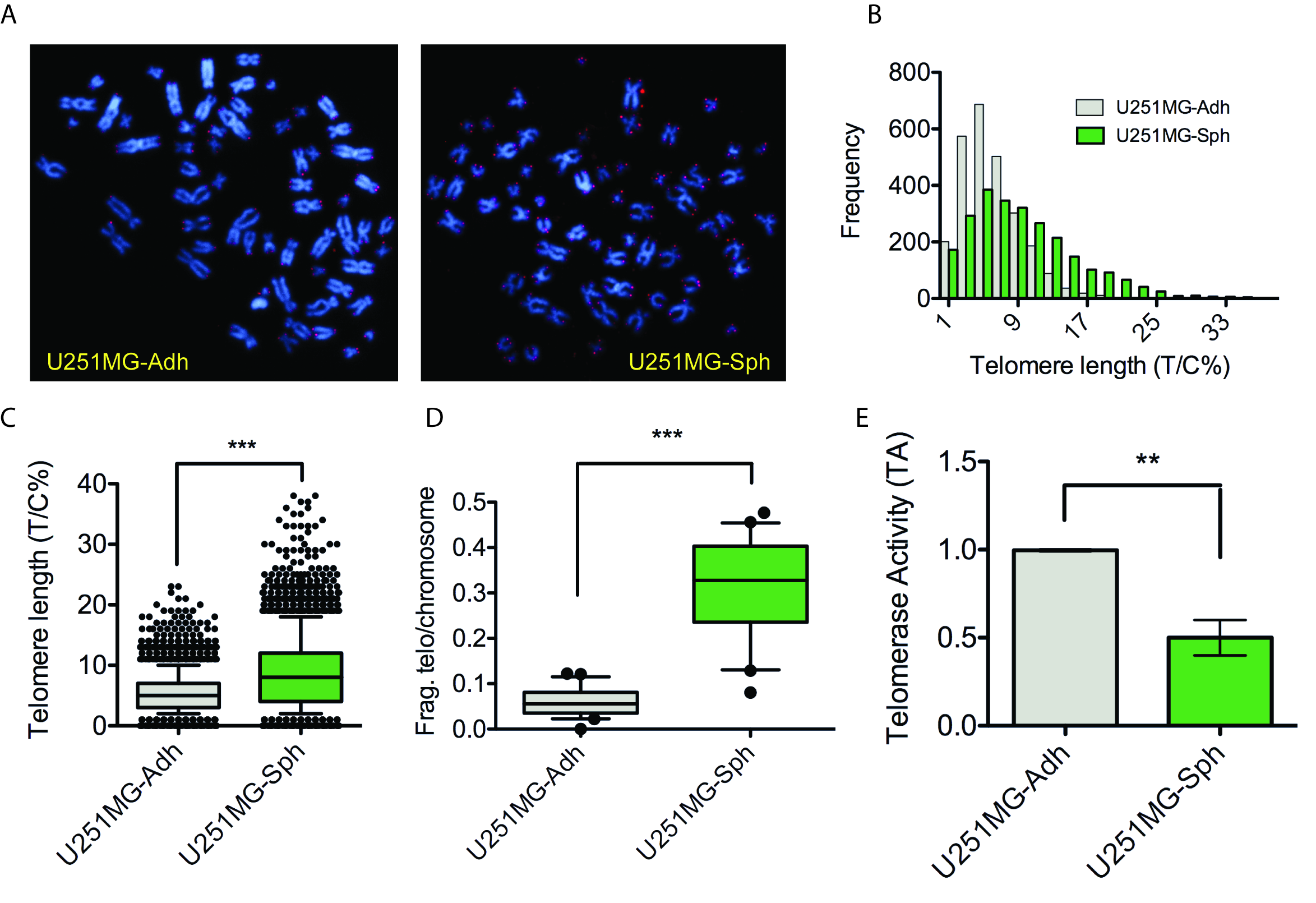

Supplement: Supplementary file 2 — Figure S2. Analysis of the telomere status in U251MG-Adh and -Sph cells. Telomere lengths were analyzed using centromere-calibrated QFISH. Representative images of metaphase spreads from U251MG-Adh and -Sph cells. (A) Distributions of telomere length in U251MG-Adh and U251MG-Sph (B and C). Box plot in C reports means, quartiles and s.d. (n = 2). Fragile telomere frequency in U251MG-Adh and U251MG-Sph. Box plot reports means, quartiles and s.d. (n = 2) (D). Telomerase activity (TA) in both parental and stem-like derived cells. Data represent mean values ± s.d. (n = 2) (E). ** P < 0.01, *** P < 0.001 (Student’s t-test). (TIF 17312 kb) [file 13046_2019_1293_MOESM2_ESM.tif]

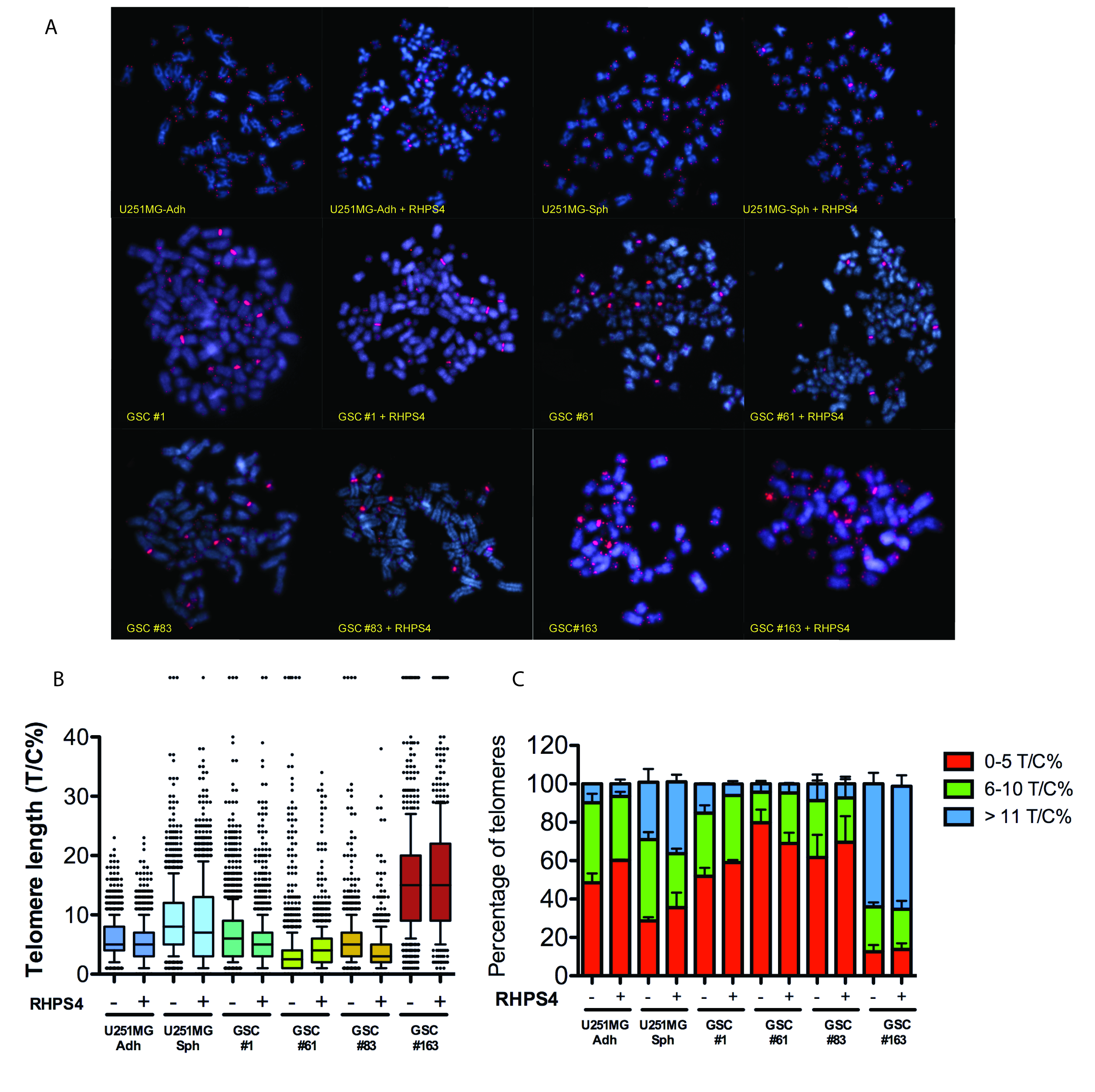

Supplement: Supplementary file 3 — Figure S3. Telomere length analysis in cancer stem-like cells treated with RHPS4. Telomere lengths were analyzed using centromere-calibrated QFISH. Representative images of metaphase spreads from U251MG-Adh and -Sph cells (A). Box Plot of telomere lengths in untreated and RHPS4 treated U251MG-Adh, U251MG-Sph, GSCs#1, #61, #83 and #163. Box plot represents means and quartiles, whiskers represent s.d. and points represent outliers. (B) Percentage of telomeres shorter than 5 T/C% (red bars), comprised between 6 and 10 T/C% (green bars) and longer than 11 T/C% (blue bars) as evaluated in untreated and RHPS4 treated cells (C). (TIF 30408 kb) [file 13046_2019_1293_MOESM3_ESM.tif]

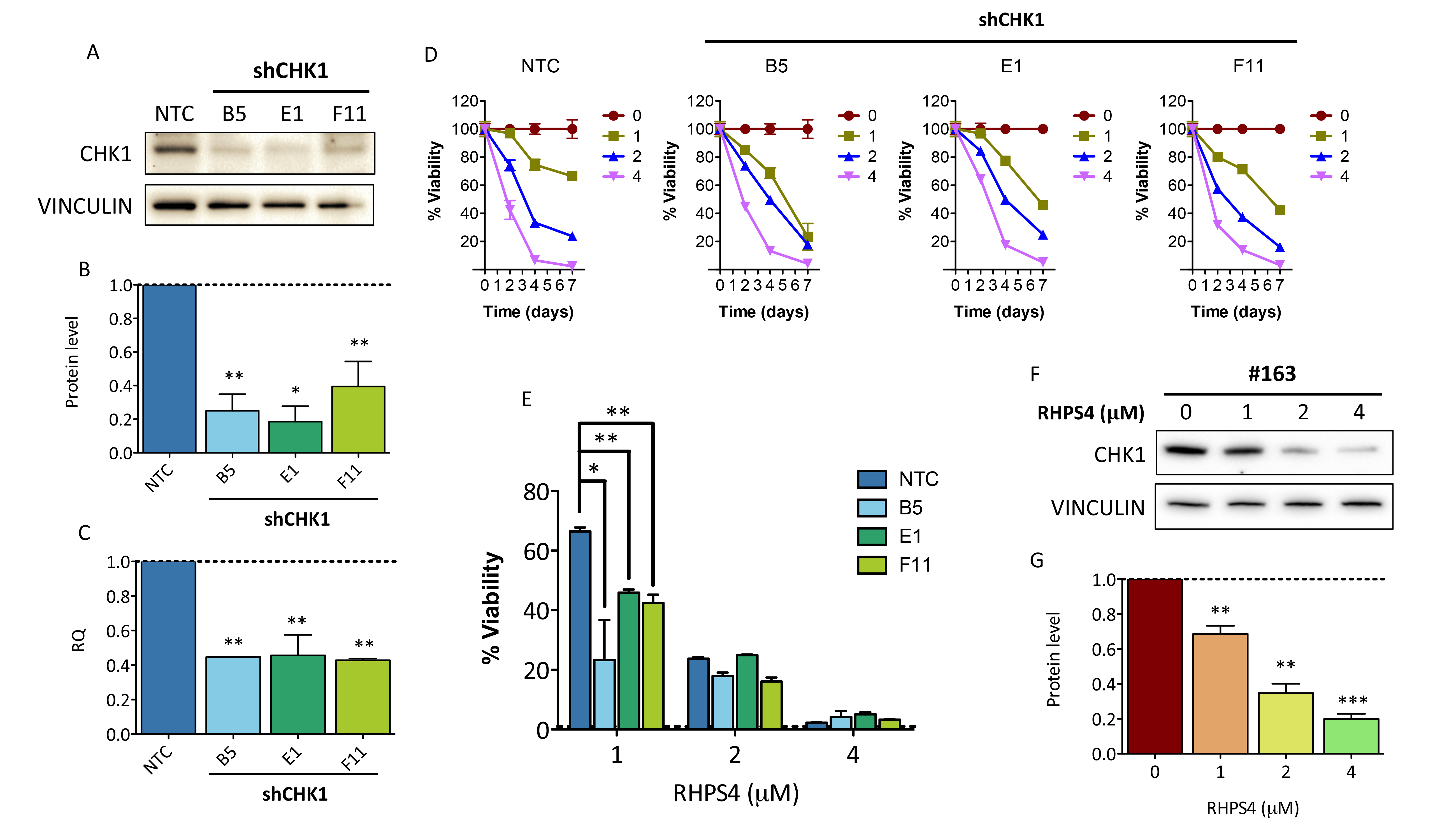

Supplement: Supplementary file 4 — Figure S4. Silencing of CHK1 increases GSC response to low concentrations of RHPS4. Protein levels of GSC #163 stably expressing either a non-targeting control shRNA (NTC) or three different shRNAs targeting human CHK1 (named shCHK1 B5, E1 and F11) are shown (A). Densitometric analysis confirmed the significant reduction of CHK1 protein levels in shCHK1 cell lines (B) and a similar reduction was also observed by means of qRT-PCR (C). Growth curves showing the effect of RHPS4 treatment (1, 2, and 4 μM) in both NTC and shCHK1 cells evaluated up to 7 days (D). Cell viability at day 7 from treatment suggests a significant impairment of cell growth in all shCHK1 cells only after 1 μM RHPS4 (E). Conversely, higher concentrations (2 and 4 μM) do not affect cell viability (E). Indeed, RHPS4 treatment in the GSC #163 is able per se to downregulate the levels of CHK1 in a dose-dependent manner (F and G) masking the difference in cell viability between NTC and shCHK1 cells after higher RHPS4 concentrations (2 and 4 μM). Data represent mean values ± s.d. (n = 2). * P < 0.05, ** P < 0.01, *** P < 0.001 (Student’s t-test). (TIF 955 kb) [file 13046_2019_1293_MOESM4_ESM.tif]

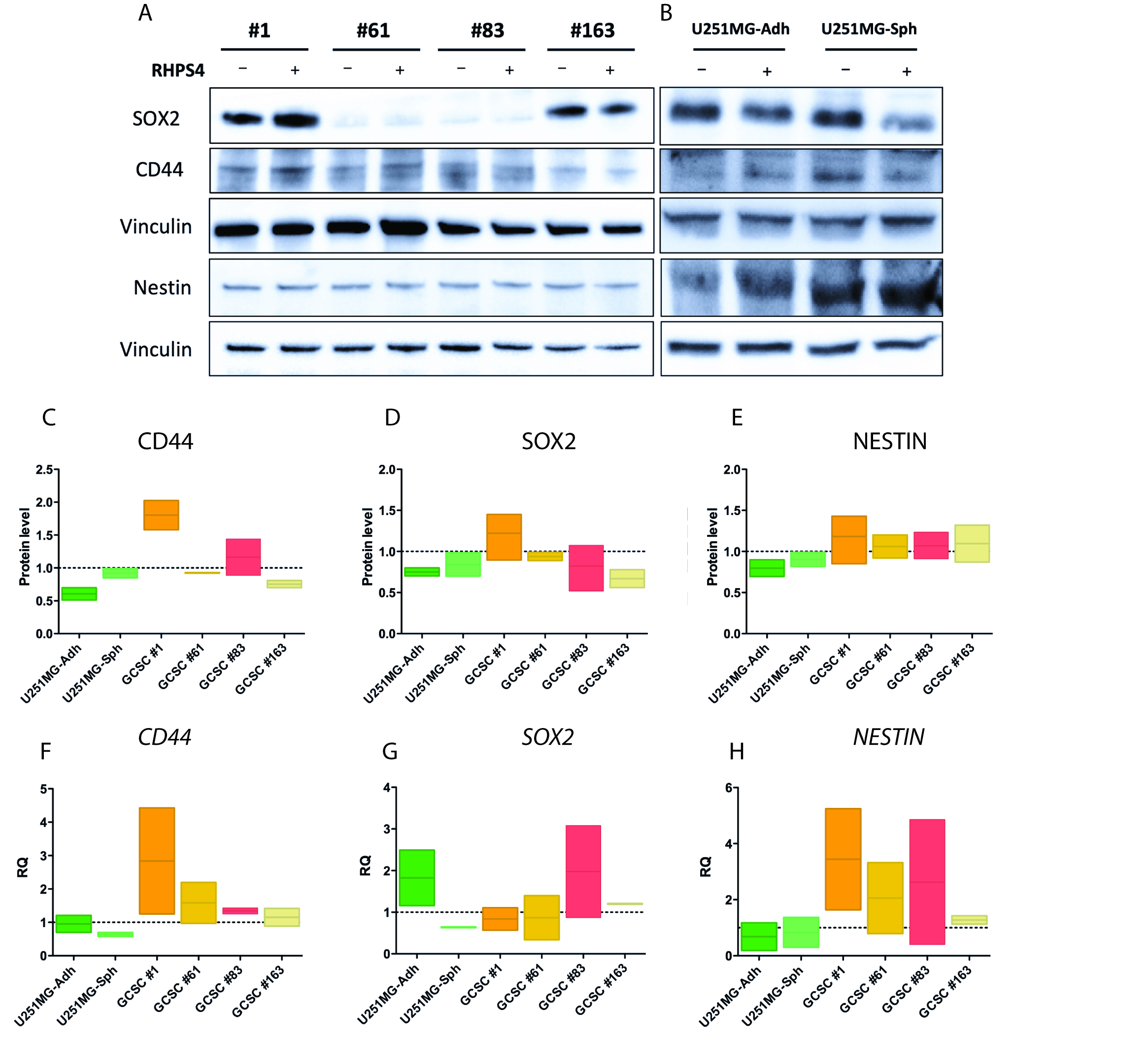

Supplement: Supplementary file 5 — Figure S5. RHPS4 does not affect stem cell markers in U251MG-Sph and GSCs. Western blot analysis of stemness markers SOX2, NESTIN and CD44 in response to RHPS4 treatment in GSCs#1, #61, #83 and #163 (A) and U251MG-Adh and U251MG-Sph cells (B). Protein levels of CD44 (C), SOX2 (D) and NESTIN (E). Box plot represents mean, maximum and minimum values (n = 2). Gene expression profile for CD44 (F), SOX2 (G) and NESTIN (H). Box plot represents mean, maximum and minimum values (n = 2). (TIF 21948 kb) [file 13046_2019_1293_MOESM5_ESM.tif]
